# Patient education for chronic musculoskeletal pain: a scoping review of recommendations, effectiveness, and educational content

**DOI:** 10.1186/s12998-025-00614-y

**Published:** 2026-01-02

**Authors:** Alice Kongsted, Anders Christer Larsen, Mette Holz Meinhardt Gregersen, Tonny Elmose Andersen, Morten Hoegh, Per Kjaer, Anne Møller, Sophie Lykkegaard Ravn, Søren T. Skou, Jan Hartvigsen

**Affiliations:** 1 Center for Muscle and Joint Health, Department of Sports Science and Clinical Biomechanics, University of Southern Denmark, Odense M, Denmark; 2 Chiropractic Knowledge Hub, Odense M, Denmark; 3 Hospital Pharmacy Funen, Odense University Hospital, Odense C, Denmark; 4 Department of Public Health, University of Southern Denmark, Odense M, Denmark; 5 Department of Psychology, University of Southern Denmark, Odense M, Denmark; 6 Department of Health Science and Technology, Aalborg University, Gistrup, Denmark; 7 Unit for Quality and Modernisation, Copenhagen S, Denmark; 8 Section of General Practice, Department of Public Health, University of Copenhagen, Copenhagen K, Denmark; 9 Research Unit for General Practice Slagelse/Køge and Copenhagen, Copenhagen K, Denmark; 10 Specialized Hospital for Polio and Accident Victims, Rødovre, Denmark; 11 The Research and Implementation Unit PROgrez, Department of Physiotherapy and Occupational Therapy, Næstved, Slagelse and Ringsted Hospitals, Slagelse, Denmark

**Keywords:** Back pain, Evidence gaps, Musculoskeletal pain, Neck pain, Osteoarthritis, Patient education, Patient preference, Practice guidelines

## Abstract

**Background:**

Patient education is considered a core aspect of health care, aimed at helping individuals understand and manage their conditions. However, no systematic overview across patient education approaches exists that can guide the optimization of educational strategies for musculoskeletal disorders.

**Objectives:**

This scoping review aimed to map 1) recommendations provided about patient education in clinical practice guidelines (CPGs), 2) research on the effectiveness of patient education, and 3) educational content recommended for chronic spinal pain, osteoarthritis, and mixed chronic primary musculoskeletal pain.

**Methods:**

Following JBI guidance for scoping reviews, we searched MEDLINE, Embase, and PsycINFO from January 2014 to July 2025 for systematic and scoping reviews, Delphi-studies, and consensus studies on patient education. Study selection and data extraction were conducted by pairs of reviewers. Findings were summarized within and across evidence types.

**Results:**

Out of 4,243 unique records screened, 178 full-texts were assessed, and 66 papers included. CPGs consistently recommended patient education, and patients emphasized a need for individualization. Educational themes included helping people understand their condition and factors impacting pain, providing reassurance, and guiding management strategies. There was substantial uncertainty about the effectiveness of various types of patient education, and no reviews investigated individualized education.

**Conclusions:**

Patient education is central in recommendations of care for musculoskeletal pain disorders, but effectiveness is uncertain, and no specific approach has been shown to be superior. We identified a gap between patient preferences for education individually tailored in a collaborative process and effectiveness studies focusing on pre-defined patient education programs.

**Supplementary Information:**

The online version contains supplementary material available at 10.1186/s12998-025-00614-y.

## Introduction

Chronic pain affects physical and mental health including people’s daily functioning, quality of life, social participation, and ability to work [1]. A substantial proportion of chronic pain is attributed to musculoskeletal disorders (MSD), which accounted for approximately 6% of global disability adjusted life years (DALYs) in 2021, with low back pain (LBP), neck pain, and knee osteoarthritis (OA) being responsible for the largest part of this burden [2].

Helping patients understand their condition and what they can do to manage it is a core aspect of MSD management [3, 4]. In this context, patient education is a structured intervention with the potential to affect how patients understand, react to, and manage their condition and thus goes beyond general advice provided as part of a clinical encounter [3].

Although patient education is considered a core aspect of care, people seeking care for MSD report that their needs for understanding their condition and available management option are unmet [5–8], and patients have pointed to education as a top priority for MSD research [9]. In addition, healthcare professionals face challenges in explaining the complexities of MSD, often struggling to move beyond structural, biomechanical, or functional goals and explanations [10–14]. Thus, patients encounter a plethora of different, sometimes conflicting and even flawed, explanations regarding their condition, which leads to confusion and frustration about what sources of information they can trust [6, 7].

In response to this need, various education programs have been developed and tested. However, there is no comprehensive status on this evidence to inform what content of patient education should be provided to patients. Also, it is very challenging to compile existing evidence across various types of patient education and different MSDs.

We conducted a scoping review to systematically map 1) the recommendations provided about patient education in clinical practice guidelines (CPGs), 2) the research conducted on effectiveness of patient education, and 3) the educational themes and messages recommended by and for people with chronic spinal pain, knee pain, hip pain, and for mixed chronic primary musculoskeletal. The aim was to inform the content of knowledge provision in patient education for adults with these MSDs.

## Methods

### Study design and registration

We followed the JBI methodological guidance for scoping reviews [15]. The protocol was registered prospectively at the Open Science Framework on 2024-04-24 (https://osf.io/gjnrq/) and the study was reported in accordance with the Preferred Reporting Items for Systematic reviews and Meta-Analyses extension for Scoping Reviews (PRISMA-ScR) [16].

### Eligibility criteria

The population of interest was adults with chronic (> 3 months duration or described as chronic) knee or hip pain with or without osteoarthritis, back pain, neck pain including whiplash associated disorders, and populations with mixed chronic musculoskeletal pain. We did not include surgical populations. To be included, studies had to be conducted with the aim to synthesise effects of patient education, synthesize clinical practice guideline (CPG) recommendations, or provide guidance on or recommendations for the content of patient education. Patient education was defined as any advice or information (verbal, written, or audiovisual) provided within a health care context with the aim of improving the patients’ understanding of their condition and/or what they could do to manage it. It could be delivered by any mode (e.g., individually or in groups, face to face or using eHealth solutions) and as stand-alone interventions or in combination with other types of care, if effects of patient education could be isolated from other parts of the intervention. We included published, peer-reviewed systematic reviews, meta-analysis, network meta-analysis, qualitative literature synthesis, Delphi-studies, and consensus statements. Best practice recommendations and overviews not based on above mentioned methodologies, commentaries, and discussion papers that were not based on a consensus process, papers focusing on specific minority groups, and dissertation and conference abstracts were excluded.

### Information sources and search

MEDLINE, Embase, and PsycINFO were searched via OVID for studies published in English and Scandinavian languages from January 2014 to March 15, 2024 and updated on July 14, 2025. We included papers published within a 10-year period as these were considered to reflect the current understanding of chronic pain. The search strategies were developed with assistance from an information specialist from the University of Southern Denmark Library and refined after being piloted and reviewed by the author team. The search strategies are available from Supplementary file 1. We further included a systematic review relevant for our scoping review not identified in our search [17], which was identified from backward citation tracking of the WHO guidelines on low back pain [3]. Identified publications were exported to Covidence, and duplicates were removed.

### Study selection

First, titles and abstracts were screened independently by two reviewers (ACL or AK plus another team member). Discrepancies were discussed after each member had screened 5–20 publications, and the criteria were clarified before completing the screening. Next, full text publications were screened by pairs of any two team members. Disagreements on selection were resolved by consensus in the pairs, involving a third reviewer if needed. Reasons for exclusions were noted for the latter screening step.

### Data extraction

A data extraction form was drafted by AK and MHMG, revised by the team, and finally refined after each team member had extracted data from at least one publication using the form. The final form included separate items for systematic reviews of primary studies, reviews of CPGs, and for Delphi and consensus studies (Additional file 1). One reviewer charted the data, and another team member checked the extraction and added any additional relevant information identified without conferring with the author who did the initial extraction. Disagreements that would lead to corrections of extracted information were solved in the reviewer pair. No attempt was made to contact study authors for information not available in publications.

### Critical appraisal

We did a general assessment of the methodological quality of the included studies, as the scoping review was intended to inform the development of educational content, and the usefulness for that purpose would depend on the trustworthiness of the evidence. Based on a list of domains to consider for the assessment (Supplementary file 2), we indicated in the extraction form if we had concerns with the applied methodology.

### Synthesis of the results

The method for the synthesis of the extracted information was dynamically developed as it was informed by the content identified in the included studies. Templates for the syntheses were outlined by AK and PK and refined with input from the author team. AK drafted the synthesis, which was then collaboratively refined through discussions with the team to ensure comprehensive integration of the data. The final synthesis was reviewed by all authors, with the tabulated extractions available for reference to maintain accuracy and consistency.

The results from systematic reviews of CPGs were synthesized in an overview of the included CPGs for each MSD, the general recommendations made in CPGs on the use of patient education, and any specific recommendations regarding the content or type of patient education. Themes of recommended content were identified across different conditions to highlight commonalities.

Information from systematic reviews of randomized controlled trials (RCTs) was synthesized to create an overview of the most frequently investigated outcomes, categorized by educational approach and condition. We reported evidence on effectiveness based on the conclusions of the included studies to inform if patient education had shown a potential to affect the outcome.

Evidence from Delphi studies and from reviews that explored patients’ perceived needs regarding education was compiled by listing the identified themes and providing examples through citations. The summary of patients’ perceived needs was conducted by first categorizing the extracted text from the studies using the ‘Thematic Analysis Generator’ on galaxy.ai running six repetitions to ensure stability of findings [18]. AK reviewed and listed the themes identified and AM then verified the face validity of the themes, and the summary provided in the result section based on these themes.

### Deviations from protocol

The protocol had planned for the entire team to pilot the screening of records by reviewing a sample of 20 titles/abstracts. However, in practice, each author did a pilot of between 5 and 20 of their assigned records and discussed any disagreements with AK and/or ACL for resolution before continuing the abstract screening.

### Patient and public involvement

Patient representatives were not involved in the planning or conducting the sëcoping review. All members of the research team have clinical experience representing chiropractic, physiotherapy, general practice, and psychology. In the next phase of our project, the findings of the scoping review will inform the development of key educational messages in a collaborative process between patients, clinicians, and researchers.

## Results

### Study selection

**Fig. 1:**
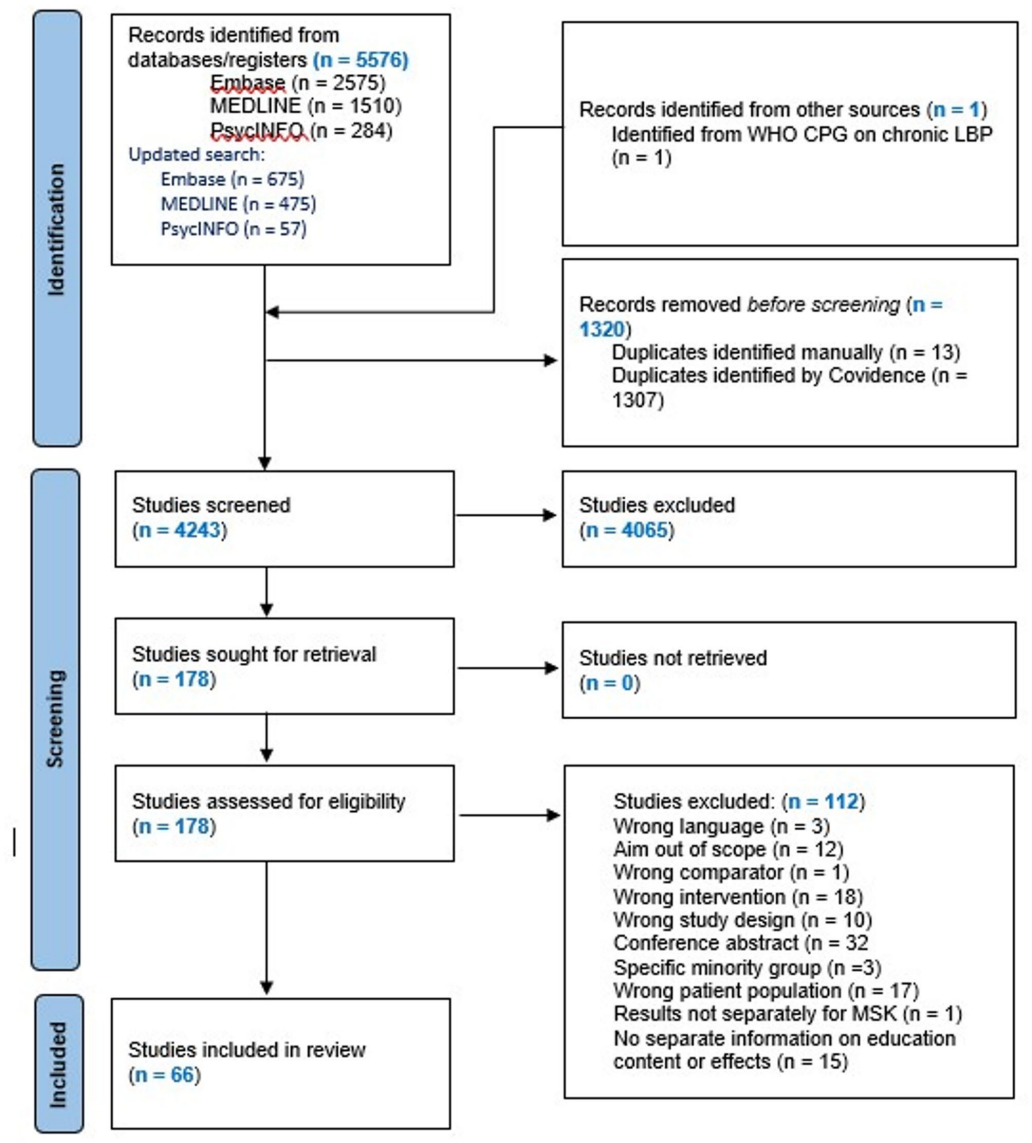
PRISMA chart of the literature search and selection

We screened 4243 records after removal of duplicates, assessed 178 in full text, and included 66 papers (Fig. 1). The included studies were 11 reviews of CPGs (LBP n = 3, any spinal pain n = 1, OA n = 6, mixed MSD n = 1) [19–29]; 40 systematic reviews of randomized trials (LBP n = 12, neck pain n = 3, any spinal pain n = 3, OA n = 11, mixed MSD n = 11) [17, 30–68]; 1 systematic review (LBP), 1 meta-ethnography (mixed MSD) [69, 70], and 1 qualitative synthesis (mixed MSD) [71] of patients’ perspectives; 2 umbrella reviews (mixed MSD n = 2) [72, 73]; 6 scoping reviews (LBP n = 3, neck pain n = 1, OA n = 1, mixed MSD n = 1) [5, 74–78]; 2 secondary analyses of systematic reviews (OA n = 1, mixed MSD n = 1) [79, 80]; and 2 Delphi studies (LBP n = 1, OA n = 1) [81, 82]. One of the systematic reviews of RCTs also included a systematic synthesis of qualitative studies [58]. Study details are available from Supplementary file 3–5. Studies excluded in full text screening are listed in Supplementary file 6.

### Certainty of evidence

We had methodological concerns for 7/11 reviews of CPGs [19, 20, 22, 23, 25–27], 29/40 of the included systematic reviews [30–33, 35, 36, 38–40, 42–45, 48, 49, 51, 53–57, 59, 60, 62, 66–68, 83, 84], 4/6 scoping reviews [5, 74–76], and both umbrella reviews [72, 73]. For some of the studies, we had only minor concerns that related to lack of pre-registration or no search in grey literature (Supplementary file 3-5). The umbrella reviews, both on pain neuroscience education (PNE) for chronic MSD, included 8 and 16 overlapping systematic reviews respectively and concluded that the quality of systematic reviews or heterogeneity across studies hindered firm conclusions [72, 73].

### Definitions of patient education

Studies that included a mix of approaches to patient education often did not provide a clear definition of the concept. When defined, patient education was typically described in general terms as education, advice, or information provided with the intention to improve a patient’s knowledge, understanding of pain and management, and enable patients to make informed decisions about their health-related behaviors [17, 37–39, 42, 62]. See Supplementary file 3–5 for details.

Studies investigating specific approaches to patient education focused on PNE, self-management education, or coping skills education. Below, we present definitions of these approaches based on commonalities across included studies.

PNE was commonly described as an approach aimed at altering patients’ understanding of pain by educating them about neurophysiological aspects and factors affecting pain, emphasizing that pain results from nervous system sensitization rather than tissue damage. The goal of PNE was to reconceptualize pain as less threatening and thereby improving patients’ ability to live well with pain, manage it, and/or reduce pain intensity through nervous system desensitization [30, 33, 34, 41, 44, 52, 54–57, 63, 72–74, 76, 79]. A scoping review concluded that PNE “does not solely use cognitive strategies; it can also adopt experiential learning techniques […] and reinforce biological concepts”, and that “PNE can be an overall approach to care that it cannot be applied without considering the corresponding theoretical model.” [64]. Illustrating that this approach has been adopted in many ways, a scoping review reported that “great heterogeneity [exists] in the teaching–learning strategies, number of treatment sessions, total duration of treatment, delivery format, content delivery modalities, and support materials” [62].

Self-management education was investigated in two systematic reviews on OA [45, 46]. The approach was defined as educational programs “focusing on education about knee OA, OA self-management or self-care, and pain coping skills, as well as self-management of diet” [45] or as programs with elements that address self-management, which "may include fostering skills in managing OA, such as problem solving, goal setting, decision making, self‐monitoring and coping with the condition, as well as providing interventions to manage pain or improve physical and psychological functioning" [46]. A review describing the content of self-management interventions for knee OA found that educational components were related to disease knowledge, treatment options, symptom management, medication management, psychological management/relaxation, and principles of self-management [60]. This was often combined with exercises, physical activity, pacing, weight management, and joint protection, often making them multicomponent interventions.

One study focused on coping skills education and defined this as a cognitive behavioral intervention, designed to help patients cope with pain by self-regulating thoughts, feelings, and behaviors [47].

### Findings from reviews of clinical practice guidelines

Eleven systematic reviews of CPGs were identified which summarized recommendations for the management of knee and hip OA, LBP, spinal pain, and mixed chronic MSD (Table 1). They consistently reported that CPGs provided moderate to strong recommendations for patient education, and patient education was among the interventions that CPGs for LBP most consistently agreed upon [52]. The CPGs recommend that patient education should be person-centered, based on individual understandings and beliefs, and targeted to the individual’s needs. Common themes included helping people understand their condition, promoting being active, providing information on self-management strategies, and addressing potential concerns, expectations, and misconceptions. In addition, CPGs for OA, recommended information about ergonomics and/or joint protection. Further, CPGs recommended that patients should be educated about their prognosis, medication (benefits and harms), and how to stay at work (Table 1).

**Table 1:** Overview of reviews of Clinical Practice Guidelines (CPGs)

MSD Author, year	General recommendation regarding patient education	Recommendations regarding type or content as summarized in reviews (theme that the statement contributed to)
LBP [21, 22,25, 26, 29, 86]	All included guidelines for chronic LBP were in favour of patient education when providing a recommendation Recommendations for individualization: Offer interventions based on patients’ clinical and psychosocial assessment findings Provide education and ongoing support for self-management that is tailored to people’s needs Provide targeted advice	Education including advice and information promoting self-management (SM) Evidence-based information on expected course (P) and effective self-care options (SM) Brief educational interventions for short-term improvement, and advice to stay active or make an early return to activities as tolerated (A) A patient with low back pain Is provided with information about their condition (C) Receives targeted advice to increase their understanding and address their concerns and expectations (CEM) Self-management is discussed with the patient because it will differ for each patient (SM)
Spinal pain Lim [28]	“Patient education was a common recommendation in all spine CPGs, despite the acknowledged low quality of supporting evidence” Non-specific LBP: 3 out of 4 CPGs recommended patient education; 1 was inconclusive Non-specific NP: patient education not considered WAD: 4 out of 4 CPGs did not recommend 'patient education alone'; patient education as part of care not considered	NA
Knee or/and hip OA [23, 27, 28]	Most CPGs had strong recommendations for patient education Patient education was recommended as core nonpharmacological intervention	Promotion of self-management strategies Information about The condition (C) Benefits of exercises (A) Lifestyle modifications (SM) Joint protection (EJ) Treatment goals (SM) Fitness and exercise goals (A) OA misconceptions (CEM) Medication effects and side effects (M)
OA mixed [20, 24, 25]	Most CPGs had strong to moderate recommendations for patient education Recommendations regarding delivery: Patient education should be an ongoing intervention Patient education should be patient centered Regular contacts should be offered to promote self-care	Information including: Ergonomic principles (EJ) Management options Individualized self-management strategies (SM) Understanding of OA (C) Education and training in exercise therapy (A) Pacing (SM) Assistive devices (EJ) Joint protection strategies (EJ)
Chronic MSD 21	Recommendations regarding delivery: Use a compassionate, patient‐centred approach for the assessment and management Explore the patient's beliefs, knowledge and understanding of pain and pain management Use interprofessional collaboration Develop an individualized and comprehensive plan of care based on the biopsychosocial model	There is (some) evidence for Addressing the patient's concerns and beliefs (CEM) Teaching the person, their family and caregivers about pain management strategies (SM) Educating patients about the risks and benefits of medications and to monitor and manage side‐effects (M) Providing advice to stay active in addition to exercise therapy rather than as a stand-alone intervention (A) Brief education to help patients continue to work (W) Self‐management strategies and self‐management resources to be provided with other therapies to ensure active patient Participation (SM)

() indicate the theme that each recommendation contributed to. (A) Being active; (C) Condition; (CEM) Concerns, expectations, and misconceptions; (EJ) Ergonomics or joint protection; (M) Medication; (P) Prognosis; (SM) Self-management; (S); Work(W)

### Findings from systematic reviews of RCTs

#### Conditions and educational approaches investigated in systematic reviews of RCTs

PNE was the approach most often investigated in the systematic reviews. Specifically, PNE was the topic of 21 (mixed MSD n = 10, LBP n = 5, neck pain n = 2, any spinal pain n = 2, OA n = 2) out of 40 systematic reviews and of 2 umbrella reviews (mixed MSD). Four reviews, all on OA, considered self-management education [45, 46, 60] or coping skills education [47]. The other systematic reviews reported on a mix of educational approaches (Supplementary File 6, Tables S3–S6).

#### Risk of bias (RoB) and certainty of evidence reported in systematic reviews of RCTs

Thirty-six systematic reviews reported on the overall certainty of evidence. Of these, 20 reported very low to low confidence in the results [17, 30, 33, 34, 43, 48, 50, 51, 83, 85–87], or that a minority of RCTs had low RoB [36, 37, 41, 44, 47, 49, 53, 61]. Twelve systematic reviews reported moderate or high certainty of evidence for some, but not all, investigated outcomes or that most of the included RCTs had low RoB [31, 35, 38, 39, 46, 54, 55, 58, 65–68], and four reported the included studies to be of “good quality” without a formal grading of evidence [40, 42, 56, 62].

#### Investigated outcomes and reported effectiveness

**Fig. 2:**
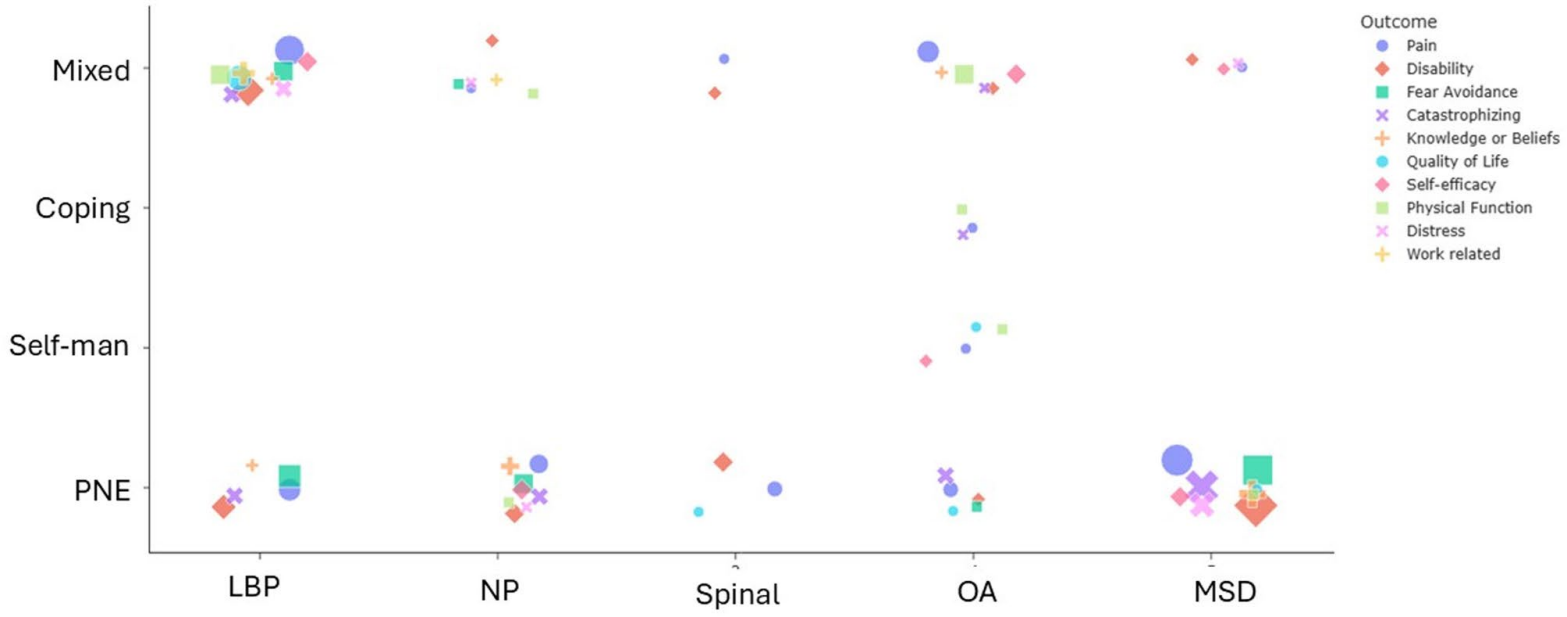
Systematic reviews by patient education approach and condition. Symbol sizes indicate the number of reviews that investigated the most frequently investigated outcomes. PNE: Pain Neuroscience Education; Self-Man: Self-management Education; Mixed: Mixed Education; LBP: Low Back Pain; NP: Neck Pain; OA: Osteoarthritis; MSD: Musculoskeletal Disorders

A wide range of outcomes were investigated, with pain (n = 35), disability (n = 26), kinesiophobia or fear avoidance (n = 19), and pain catastrophizing (n = 17) being the most frequent. Nine reviews, including seven on PNE, included an outcome capturing effectiveness on knowledge or beliefs (Fig. 2 + Supplementary file 6, Tables S3–S6).

Findings regarding the effectiveness of patient education were mixed. Thirty-four out of 40 systematic reviews concluded that patient education had positive effects for one or more outcomes. As previously noted, the certainty of evidence was low, and findings were often inconsistent across outcomes and follow-up time points (Supplementary file 5). Reported findings of systematic reviews were generally in favor of patient education in combination with other elements, most often exercises [17, 31, 33, 37, 37, 40, 42, 48–50, 55, 68] (Supplementary file 5). Also, a large network meta-analyses of mixed patient education interventions [37] and an umbrella review of PNE [72] demonstrated larger effects when education was combined with other interventions. Another umbrella review on PNE concluded that it was not possible to make firm conclusions based on available evidence [73]. In the only systematic review on pain coping skills training, effect sizes did not reach the predefined minimal important difference, and pain coping education was found equally effective with and without exercises [47].

None of the identified systematic reviews conducted a head-to-head comparison of different patient education approaches. One subgroup analysis that isolated PNE from other approaches did not identify substantially different outcomes with that approach as compared to patient education in general [17]. None of the studies provided evidence to determine whether specific content or messages are important for the effectiveness of patient education. One study planned to investigate how elements of PNE contributed to treatment effects but concluded that there were not enough studies to conduct this sensitivity analysis [63].

#### Dose and mode of delivery

Four systematic reviews reported on preplanned moderation or sensitivity analyses to determine the most effective dose or mode of delivery for patient education [17, 31, 41, 57], and one reported that the planned analyses could not be conducted due to heterogeneity in reporting [37].

The dose of patient education varied substantially, from delivered in a booklet or in a 5-min session, to sessions lasting more than an hour and comprehensive programs lasting up to one year (Supplementary file 5). The results concerning the dose of patient education mostly indicated larger improvements with higher dose [31, 34, 41], although one moderation analysis did not support this [57]. A systematic review of minimal interventions consisting of just one session suggested that this was no more effective than no intervention and slightly inferior to other interventions such as exercises, manual therapy, or more comprehensive patient education [38]. Systematic reviews, aiming to investigate the dose–effect relationship, estimated that 100 to 400 min of PNE are necessary to achieve a minimal clinically important difference, depending on the outcome measure [30, 54].

Results concerning the delivery format of patient education were inconsistent. Results were reported both in favor of group-based [31, 57] and individual education [34], but no study investigated individualized versus generic information. Subgroup-analyses in one high-quality systematic review did not find evidence to support substantial different outcomes with different modes of delivery [17].

Data extractions on dose and delivery mode are available from Supplementary file 5.

### Findings regarding educational content from consensus papers and reviews of patients’ perspectives

Two Delphi studies combined clinician, patient, and research expertise to provide recommendations for content of patient education for LBP and OA [81, 82]. The recommended educational themes were staying active, identification of red flags, reassurance, principles of management, treatment options, unnecessary interventions, and disease knowledge (Table 2). Recommendations differed somewhat between LBP and OA with reassurance and influence of psychological and social factors described as core content only for LBP, while a structural explanation for OA “Osteoarthritis is not just a disease of the cartilage but affects your whole joint including muscles and ligaments” was provided for OA. A more biomedical approach to OA as compared to LBP was also reflected in different themes for discussing management options (Table 2).

**Table 2:** Overview of evidence from consensus papers

MSD Author, year	Panelists	Themes	Messages
LBP French [70]	Chiropractors General practitioners Medical doctors Patients / consumers Physiotherapist	Stay active Red flag identification Reassurance Unnecessary interventions Principles of management Disease Knowledge	Stay active When you have back pain, staying active is important. You need to pace yourself to return to your usual activities When you have back pain, carry on with normal activities as far as possible Staying active helps prevent long-term back problems Bed rest for more than a day or 2 is not good Do not take back pain lying down Red flag identification You should see a health practitioner URGENTLY if you have back pain and either of the following: bladder and/or bowel disturbance, significant leg muscle weakness You should see a health practitioner if you have back pain and any of the following: pain that spreads down one or both legs; a fever; recent invasive surgery; recent significant trauma; unexplained weight loss; history of cancer You should see your health practitioner if your back pain is severe and it is worrying you, if you are having difficulty managing your back pain, or your pain is getting worse Reassurance Your pain may not necessarily be related to the extent of damage in your back. Hurt does not necessarily mean harm Most people find that their back pain settles down over a short period. If your back pain persists and is worrying you, consult a health professional In most cases of recent onset back pain, the pain will get better in several weeks; However, this varies from person to person It is rare for low back pain to be caused by a more serious health problem Most people have pain in their low back at some stage in their lives It is not necessary to know the specific cause of your back pain to manage the pain effectively It is normal to worry about the cause of your back pain and the impact it may have on you Unnecessary interventions Imaging (eg, x-ray, computed tomography [CT] scan, or magnetic resonance imaging [MRI]) is usually not needed in the majority of cases of low back pain, particularly when your pain has been present for less than 6 weeks. Talk to your doctor about this X-rays will not highlight the cause of pain in most cases unless a fracture is suspected CT scans have little use in diagnosing back problems and caution should be exercised due to the large amount of radiation involved with their use Blood tests are usually not needed in the majority of cases of low back pain Principles of management Work towards returning to your usual activities, with guidance from your health practitioner Persistent low back pain is influenced by a number of factors—physical, emotional, and environmental; so, it is important to address each of these areas Take ownership of your own well-being Staying positive is important. Help is available Work with your health care team to set goals Work with your health practitioner to address your concerns Work with your health practitioner to manage your back pain If you have any further questions to ask your health practitioner, write them down and discuss them at your next visit Health practitioners can assist in screening for causes of back pain Disease knowledge In around 95% of cases, it is not possible to pinpoint the cause of back pain Low back pain may happen again over time
Hip and knee OA French, [69]	General practitioners Orthopaedic surgeons Patients /consumers Physiotherapists Researchers	Disease knowledge Principles of management Exercise, physical activity and weight loss Drugs Surgery	Nondrug treatments have similar benefits for your osteoarthritis symptoms to pain relieving drugs, but with very few adverse side effects Actively taking part in self-management programs could benefit your osteoarthritis Treatment interventions and lifestyle changes for your osteoarthritis should be individualized and include long- and short-term goals Goals should be reviewed regularly with your health professionals Methods for you to self-manage your osteoarthritis should be discussed and agreed on by you and your health professionals Osteoarthritis is not just a disease of the cartilage but affects your whole joint including muscles and ligaments Joint damage on an x-ray does not indicate how much your osteoarthritis will affect you The symptoms of osteoarthritis can vary greatly from person to person Osteoarthritis is not an inevitable part of getting older Regular physical activity and individualized exercise programs (including muscle strengthening, cardiovascular activity, and flexibility exercises) can reduce your pain, prevent worsening of your osteoarthritis, and improve your daily function If you are overweight and have osteoarthritis, it will be beneficial to lose weight and maintain a healthy weight through an individualized plan involving dietary changes and increased physical activity Living a sedentary life could worsen your osteoarthritis and also increases your risk of other lifestyle-related diseases Individualized exercise is an integral component of treatment for everyone with osteoarthritis Maintaining sufficient muscle strength around the joints is important in reducing pain and maintaining function, and if you require an operation will benefit both pre- and post-operative periods of your treatment Linking your individualized exercises to your other daily activities is a useful way to become more active Individualized exercises only work for your osteoarthritis if you do them regularly Small amounts of individualized exercise undertaken frequently can be beneficial for your osteoarthritis You should avoid the use of nonsteroidal anti-inflammatory drugs for your osteoarthritis over the long term You may get some pain relief from your osteoarthritis by using acetaminophen (paracetamol) medications Your osteoarthritis symptoms can often be eased significantly without requiring an operation If you cannot achieve pain relief from your osteoarthritis, have undertaken a sustained period of recommended conservative management, and it is very difficult to perform activities of daily living, joint replacement surgery is an option Keyhole surgery (arthroscopy) that involves washout of the joint and joint scraping should not be used to treat your pain unless there is a mechanical blocking of your joint

Two studies synthesized qualitative evidence concerning patients’ perspective on participating in PNE [58, 71]. The findings highlighted that a comprehensive assessment allowing the patient to share their personal narratives should be undertaken to ensure they feel heard. Furthermore, it was emphasized that PNE should be delivered by health care professionals skilled in offering emotional support and in tailoring information to individual needs and prior understanding [58, 71] (Supplementary file 1c).

Two scoping reviews [5, 75], 1 meta-ethnography [69], and 1 systematic review [70] investigated patients’ perspectives on perceived needs for knowledge (Table 3). These considered mixed MSD, LBP, and OA. Similarly to the findings on PNE, these studies illustrated that content of the information provided is important, but its usefulness relies heavily on the patient-clinician interaction (Table 3 + Supplementary file 7).

**Table 3:** Overview of evidence from reviews of patients’ perspectives extracted from the studies

MSD Author, year	Design Number of studies / informants	Overarching themes	Patients’ needs regarding delivery and context	Patients’ needs regarding content
Chronic MSD Thompson, 2022 [69]	Meta-ethnography to inform physiotherapy training N = 18 studies N = 300 patients	An acceptable explanation for pain Professional influence/seeking hope and direction from physiotherapy Perceptions of effectiveness/speed of improvement Supported self-care/shared decision making/respect and dignity Complex emotions accompany chronic pain/supportive environment The health service ‘merry-go-round’, that is, continuous cycle of consultations repeating story Exercise—a strategy and barrier for patients who are in pain Embedded/embodied experience of pain Sharing experiences, benchmarking, validation, perceptions of effectiveness Creating new patterns of thinking and acting, behaviour change, transition in healthcare Motivating factors—intrinsic and extrinsic motivations, incentives, technology How/who makes decisions	People in pain are individuals: participants want to be listened to, believed and enabled to actively participate in a two-way consultation Pain management needs to be accessible and realistic: People living with chronic pain described needing a healthcare system that was accessible like other long-term conditions Context matters: Healthcare environments that promoted feelings of relaxation, a sense of belonging, and a safe environment to express thoughts and emotions were reported to be encouraging and motivating	What does pain mean?: information is critical to help people understand and navigate the complex nature and meaning of pain. The crucial role that physiotherapists have in facilitating conversation about pain; to analyse patient narrative to understand what explanatory model is being used by the patient to underpin the meaning of their pain Pain is complex—there is a lot to learn: Physiotherapeutic approaches that made participants more aware of the impact of thoughts and emotions on pain, activity levels and behaviour were interpreted to be positive Hope is really important—the power of health professionals: Participants sought hope, reassurance and guidance from the professional person. Physiotherapists’ role in coaching and motivating others, and fostering self-efficacy in people experiencing pain is an important concept
Chronic MSD Ciolan, 2025 [71]	Meta-synthesis of qualitative studies N = 9 studies including n = 181 participants	Efficient communication of information: Relationship and Communication, Delivery Methods, and Relevant Content and Topic Emotional support and well-being: Emotions and Feelings Empowerment promotion: Reconceptualisation of the Pain Mechanisms and Beliefs, Mindset and Coping Strategies	The importance of clear and effective communication characterised by appropriate wording and active listening Participants found videos helpful in delivering PNE Mixed about digital animations, as they saw the animations to facilitate understanding but also dehumanizing	General approval of the contents and topic of the PNE, which allowed them to understand their pain condition better “They explained it very well, because at the general practitioner I got a blue booklet about chronic pain. About nerves and how it all works. That your body is actually a burglar alarm set incorrectly. That one I remember, when people ask me how I am doing and what was discovered, I tell them that. It [the metaphor] appeals to the imagination.” Participants also reported widespread dissatisfaction with the content and materials of PNE. They indicated that they felt the content did not contribute anything new to their knowledge of pain. Important topics had not been addressed, such as the causes of pathology or specific indications (e.g. the type of diet in fibromyalgia). Others would like more information on the mechanical aspect of the problem Other participants reported a lack of relevance and usefulness resulting from the little personalisation of the PNE content to their specific situation The information received changed some participants’ ideas about pain mechanisms: “I think the most important concept for me was learning that my brain was the problem not my arm.” “My pain system is being too sensitive because of all the stress and illness in my life and worry” Participants also reported that the strategies proposed by PNE might not be very applicable in everyday life and do not give precise indications on how to deal with pain
LBP Chou, 2018 [75]	Scoping Review N = 43 studies including n = 9–133 participants	The need for good communication skills The need for shared decision-making, respect, and being listened to The need for empathy, understanding and confidence Patients want information that is understandable and informative, explained in “layman's terms.” Communication style should be encouraging and personalized to the individual Both treatment and delivery of treatment to be individualized		Patients had a need for a diagnosis and finding a cause of pain; excluding pathology was important Patients wished to find out about the problem, the prognosis, and how to prevent or manage recurrence so they commonly sought information on the Internet Patients wanted a diagnosis for their pain, and they felt that this was necessary to inform management and prevent recurrence Patients needed an accurate diagnosis and considered [it] to be an acceptable means of validating the individual’s distress and contributed to improved treatment outcomes Patients want a medical diagnosis but also acknowledgment that psychosocial factors contributed to their pain Patients want a diagnosis for the back pain and they hoped to meet an expert on back pain and get a clear and precise explanation for their pain Patients want to know why they had pain HCPs should integrate systematic management of sexual problems in chronic LBP consulting Patients think that getting information about treatment options from HCPs is important Patients wanted providers to give them explanations about the pain and information about coping with their pain Patients want direction, being told to rest is not only ineffective but counterproductive Doctors should help patients see which limits can be exceeded—want to know what activities they can do and how to actively get better they valued information that would help them deal with their back pain themselves and were prepared to make behavioral changes which might help alleviate their symptoms Most frequently cited area of dissatisfaction was an inadequate explanation of the problem and poor understanding of what was wrong
LBP Lim, 20189 [70]	Systematic review 41 RCTs (including 22 on chronic LBP); 3936 patients	General information related to LBP Diagnosis and cause/etiology Perceived needs for imaging Prognosis, including future disability and effect on work capacity Information regarding management of flares and preventive measures LBP management Self-management strategies Support services for LBP	A need for high quality information (reliable, updated, evidence-based, valid, consistent) Need for health information to be delivered in a suitable tone and understandable language (open, clear, emotional support, focus on personal circumstances, avoid medical terminology, show understanding towards patients) Need for credible and trusted sources (alternative sources leading to conflicting advice)	“I had just no frame of reference to figure out like what it was…with a back. I don’t know… I’m just completely in the dark” “Biomechanical and ‘anatomical’ explanation of their back problems: he must explain the wrong movements and positions…; I wanted to know what are the lumbar, coccygeal vertebrae made of and what is spondylosis” “Need imaging tests to provide reassurance and confirmation of diagnosis: Xray was to establish whether…was just a pulled muscle or whether it was a herniated disc” Need tests or imaging to confirm legitimacy of LBP: *I kind of cried with relief when I saw what was wrong… but you don’t want this unexplained pain* Important to know and gain understanding of prognosis of LBP—‘… explained that it may get worse if I continue with my bad habits but if I watch how I sit I will be fine… that was a relief’ Prognosis of LBP is important—‘… felt powerless in the face of their LBP and feared that it would be chronic…’ Participants wanted to gain self-control of the unpredictable nature of LBP, especially with flare-ups: *I’d lost confidence in my back because it can go at any time…; They’re getting fed up at work you know, when flare-up happens* *It is my back, it’s my responsibility to always look after it; …must explain the plan in steps within a timeframe and the benefits of every exercise* Need advice on how to return to normal activities Patients wanted to know the role of simple analgesia Patients desired strategies to prevent exacerbation of LBP, to reduce anxiety from the unpredictable nature of LBP Patients wanted to know about self-management, ie, what they could do about the pain and future treatment plan: *I’m crying out for somebody to take an interest in me for I’m a fighter and I want to improve my health* Need information regarding social network/support groups available. *I don’t even know where to look…; Information is just not there; it’s not available* Patients wanted information regarding absence management policy and procedures,
OA (mixed)—mainly knee and hip Chou, 2018 [5]	Scoping Review N = 30 studies including n = 5- 4478 participants	The need for clear communication of health information The need to obtain health information from a variety of sources The needs of health information content	Patients wanted sufficient time with the healthcare practitioner to explain everything They felt there was a contradiction in the advice and information given to some participants by various health care practitioners, which may indicate a lack of knowledge from the practitioner Participants expressed a strong sense of dissatisfaction with the insufficient amount of information provided Patients were dissatisfied with the perceived lack of understanding, the type of help and information received from some health care practitioners Patients felt they had to ask for health care advice, rather than be given the information spontaneously Advice and response to questions in particular about topics in the media were perceived as generally good Patients typically relied on their doctor for general medical information, but once diagnosed with OA, all participants stated they were keen to use the Internet as an alternative source of information Participants stated that despite the use of a variety of search engines, sourcing relevant and credible health information from the Internet was difficult Patients learned coping strategies from health professionals, the media, Internet, physical therapists, doctors and fellow sufferers Participants purposefully seek information about arthritis and their health status through print media, experts at classes or on television, by consulting nurses and by listening to friends Patients felt that learning from others’ experiences provided hope for a better future Patients who knew someone who had undergone similar procedures considered themselves at an advantage in being able to share their experience. Support from friends, family and significant others who had undergone similar procedures were regarded as invaluable	Patients wanted clear, easy to understand information Patients thought that practitioners were frequently not explicit enough when discussing the seriousness of the diagnosis or the value of certain drugs compared to others Participants wanted clear communication of individualised care plans Many terms used in OA are misunderstood by patients

Patients emphasized the need for clear communication and individualized information. They communicated that it was essential that health care providers validated their pain experience, showed empathy, and provided psychological and motivational support fostering hope, reassurance and realistic optimism [5, 69, 70, 75].

The studies identified a strong desire for comprehensive information about the condition and an accurate diagnosis [70, 75]. Patients considered a diagnosis crucial to serve the purpose of validating their pain, guiding management, and for their ability to communicate what is wrong to others. To understand the disease, patients both described a need for specific structural explanations and for education on the complex nature of pain, including the impact of thoughts and emotions on pain and behavior [69, 75]. Further, the studies identified a need for information about self-management, pain coping strategies, imaging and tests, and treatment options, and for being involved in the treatment planning process (Table 3).

Reliable sources of information and accessible health care were identified as another area of importance to patients. Patients wanted continuity in care and assistance to navigate the health care system. They looked for credible information about their conditions from many sources and pointed to the challenges of sorting available information (Table 3).

Content mapping of 41 educational materials revealed that only one of these addressed all eight information needs identified in an earlier review by Lim and colleagues [70, 78].

## Discussion

Our findings demonstrated wide agreement that patient education intended to facilitate helpful reactions to pain and help people navigate the health care system is a key aspect of care for people with MSD. The findings suggested that content should provide patients with knowledge about their condition, information to help understand their pain and what influences it, and information about management options with an emphasis on self-management strategies. Further, our results highlighted that patient education should be targeted to the individual patient’s needs and delivered with empathy and validating patients’ experiences. Conclusions from systematic reviews of effectiveness supported the provision of patient education for MSDs, although the evidence of effect was uncertain.

**Fig. 3:**
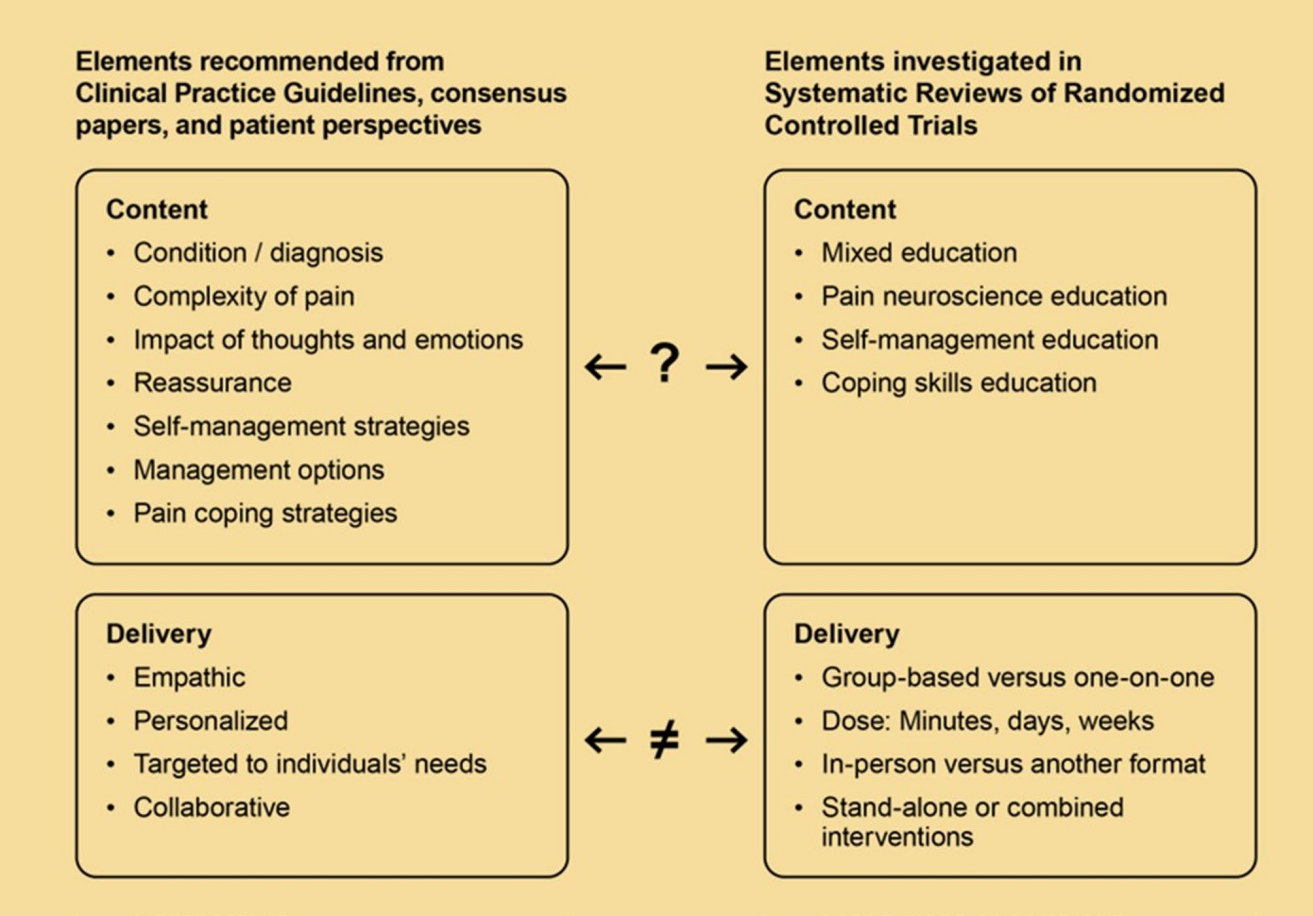
Key elements advocated in clinical practice guidelines, consensus papers, and investigations of patients’ perspectives (on the left) and elements investigated for effectiveness summarized in the systematic reviews (on the right). The “?” indicates that inclusion criteria in systematic reviews leave uncertainty with the extent to which content elements are covered in investigated patient education interventions.

There was lack of evidence comparing the effectiveness of different approaches to patient education, and besides studies on PNE, there were no descriptions of the specific content covered. Patients and other experts clearly advocated for individualized, person-centred education rather than a generic package of information. However, reviews of effectiveness studies focused exclusively on pre-defined content and none of the studies reviewed evidence on individualized versus generic interventions or considered ways to provide person-centred education (Fig. 3).

All sources of evidence pointed to the potential benefits of combining patient education with physical activity, supporting the notion that altering disease related behaviors requires both knowledge and skills. For example, CPGs recommended that patient education includes advice on staying active and/or doing exercises, consensus based on Delphi processes advocated for including education about exercises and physical activity, and patients asked for directions how to return to activity with exercises considered both a strategy and a barrier. This aligned with findings from systematic reviews of effectiveness studies that indicated education to be more effective when combined with physical elements, which were mainly exercises.

We observed some differences between research dealing with spinal pain and OA. PNE was the most investigated approach to patient education for people with LBP, whereas we only identified systematic reviews on self-management and coping skills education for people with OA. Also, the provision of knowledge regarding psychological and social factors played a larger role in recommendations for LBP than for OA. There are many similarities between LBP and OA including weak associations between pain and image findings, and management for both should be guided by symptoms and activity goals [88, 89]. Therefore, differences in educational approaches are likely based in tradition and little communication between the two research fields, pointing to potential benefits of increased knowledge-transfer between different MSD fields. The few studies on self-management education is most likely a result of the fact that they were excluded if they integrated education with other interventions [90, 91].

The main limitation of this scoping review is that it did not include primary research. Consequently, relevant studies were only considered if they were included in reviews and published prior to search dates for the included reviews. This meant we had to rely on information on intervention content provided in the reviews, which often did not include details on the specific educational content delivered. We further restricted the inclusion of systematic reviews of RCTs to those investigating patient education in isolation. As a result, reviews of interventions combining education and other elements were only included if the comparisons isolated the educational component. In addition, we excluded studies focused on eHealth delivery of patient education, as our review was limited to interventions implemented in clinical settings. This may have led to the omission of relevant evidence on digital formats that are increasingly used in practice.

Lastly, although we acknowledge the importance of adapting patient education to individual and cultural needs, we did not include studies targeting specific minority groups. We considered such adaptations of interventions to be beyond the level of detail that could be adequately addressed within the review. Together, these restrictions mean that the scoping review did not capture all evidence on patient education for the conditions investigated. However, they made it feasible to provide a broad and structured overview of the field.

Notably, our search strategy did not identify a systematic review refenced in the WHO guideline for non-surgical management of chronic primary low back pain. The most likely reason is that we searched for “education” as a subject heading but not as a free text stand-alone term. Testing showed that including “education” as a free text stand-alone term resulted in an unmanageable number of irrelevant records and excessive noise. We cannot rule out the possibility that other relevant studies may have been missed due to this.

Finally, we reported the authors’ conclusions from included studies and conducted only a general assessment of methodological quality rather than applying formal risk of bias tools or grading the certainty of evidence. Despite such quality assessment being unconventional for a scoping review, we included it to offer an indication of study quality, which may be useful when existing knowledge is used to inform the development of future patient education.

Despite a substantial body of literature, this overview calls for research that can better inform the provision of patient education for people with MSD in clinical practice. There is consensus that people with chronic MSDs should be offered education, so the main interest for future studies is not whether it is effective or not, but how it is best provided, i.e. a need for high quality trials designed to inform if some approaches and elements are more effective than others, and how education is most effectively individualized. This will include studies on how to best enhance clinician skills in engaging in person-centred care and in using respectful and effective communication techniques that foster a partnership with patients, previously highlighted as a key priority for educating pain clinicians [6]. Whereas all types of patient education intend to facilitate behaviour change in patients, it differs how this is hypothesized to be achieved. Moving forward, it would therefore be helpful to systematically describe interventions using frameworks for behaviour change and communication styles for example the ‘The Behaviour Change Intervention Ontology’  [90, 91]. Also, outcome measures capturing knowledge, cognitions, and skills should be systematically included to better understand mechanisms of effect. Furthermore, we hope that our findings will help guide future research efforts by informing the development of more narrowly defined research questions to be addressed in reviews of primary studies.

## Conclusion

Across clinical practice guidelines and systematic reviews, patient education was recommended and supported as a core element of care for musculoskeletal pain disorders, and patients placed significant emphasis on this. While the necessity of offering patients access to information about their condition and how they best manage it is undisputed, important questions remain about how best to do this.

Patients preferred education that is individualized in a collaborative process between clinicians and patients, however studies on effectiveness focused entirely on content of the information. Also, outcomes were mostly not designed to capture effects on the knowledge, cognitions, or skills directly targeted by the interventions.

There is a need for high quality research to determine what patient education approaches are most effective for whom, and for evidence to inform how evidence-based content can be matched to individual patient needs. Findings that patient education is not merely about content but also about delivery, further point to a need for evidence on ways to enhance clinicians’ skills in providing patient education.

## Supplementary Information

Below is the link to the electronic supplementary material.

Supplementary Material 1

Supplementary Material 2

Supplementary Material 3

Supplementary Material 4

Supplementary Material 5

Supplementary Material 6

Supplementary Material 7

## Data Availability

All data generated or analysed during this study are included in this published article and its Supplementary files.

## Abbreviations

CPG: Clinical practice guidelines
DALYs: Global-disability adjusted life years
LBP: Low back pain
MSD: Musculoskeletal disorders
NP: Neck pain
OA: Knee osteoarthritis
PNE: Pain neuroscience education
PRISMA-ScR: Preferred reporting items for systematic reviews and meta-analyses extension for scoping reviews
RCTs: Randomized controlled trials
RoB: Risk of bias

## References

[1] MacNeela P, Doyle C, O’Gorman D, Ruane N, McGuire BE. Experiences of chronic low back pain: a meta-ethnography of qualitative research. Health Psychol Rev. 2015;9(1):63–82. 25793491 10.1080/17437199.2013.840951

[2] Healthdata.org [Internet]. 2021 [cited 2025 Jan 20]. GBD 2021 Cause and Risk Summary: Musculoskeletal disorders - Level 2 cause. Available from: https://www.healthdata.org/research-analysis/diseases-injuries-risks/factsheets/2021-musculoskeletal-disorders-level-2-disease

[3] World Health Organization. WHO guideline for non-surgical management of chronic primary low back pain in adults in primary and community care settings. Geneva: World Health Organization; 2023. p. 1. 38198579

[4] Lin I, Wiles L, Waller R, Goucke R, Nagree Y, Gibberd M, et al. What does best practice care for musculoskeletal pain look like? Eleven consistent recommendations from high-quality clinical practice guidelines: systematic review. Br J Sports Med. 2019;54(2):79–86. 30826805 10.1136/bjsports-2018-099878

[5] Chou L, Ellis L, Papandony M, Seneviwickrama KLMD, Cicuttini FM, Sullivan K, et al. Patients’ perceived needs of osteoarthritis health information: a systematic scoping review. PLoS ONE. 2018;13(4):e0195489. 29659609 10.1371/journal.pone.0195489 PMC5901923

[6] Slater H, Jordan JE, O’Sullivan PB, Schütze R, Goucke R, Chua J, et al. ‘Listen to me, learn from me’: a priority setting partnership for shaping interdisciplinary pain training to strengthen chronic pain care. Pain. 2022;163(11):e1145–63. 35384928 10.1097/j.pain.0000000000002647 PMC9578532

[7] Smuck M, Barrette K, Martinez-Ith A, Sultana G, Zheng P. What does the patient with back pain want? A comparison of patient preferences and physician assumptions. Spine J. 2022;22(2):207–13. 34551322 10.1016/j.spinee.2021.09.007

[8] Söderlund A, Nordgren L, Sterling M, Stålnacke BM. Exploring patients’ experiences of the whiplash injury-recovery process: a meta-synthesis. J Pain Res. 2018;29(11):1263–71. 10.2147/JPR.S158807 PMC6029586 29988716

[9] Lyng KD, Larsen JB, Birnie KA, Stinson J, Hoegh MS, Palsson TS, et al. Participatory research: a priority setting partnership for chronic musculoskeletal pain in Denmark. Scand J Pain. 2023;23(2):402–15. 35918804 10.1515/sjpain-2022-0019

[10] Holopainen R, Simpson P, Piirainen A, Karppinen J, Schütze R, Smith A, et al. Physiotherapists’ perceptions of learning and implementing a biopsychosocial intervention to treat musculoskeletal pain conditions: a systematic review and metasynthesis of qualitative studies. Pain. 2020;161(6):1150–68. 31977935 10.1097/j.pain.0000000000001809

[11] Hubeishy MH, Rolving N, Poulsen AG, Jensen TS, Rossen CB. Barriers to the use of clinical practice guidelines: a qualitative study of Danish physiotherapists and chiropractors. Disabil Rehabil. 2024;46(1):105–14. 36537245 10.1080/09638288.2022.2157501

[12] Ng W, Slater H, Starcevich C, Wright A, Mitchell T, Beales D. Barriers and enablers influencing healthcare professionals’ adoption of a biopsychosocial approach to musculoskeletal pain: a systematic review and qualitative evidence synthesis. Pain. 2021;162(8):2154–85. 33534357 10.1097/j.pain.0000000000002217

[13] Nissen N, Holm PM, Bricca A, Dideriksen M, Tang LH, Skou ST. Clinicians’ beliefs and attitudes to physical activity and exercise therapy as treatment for knee and/or hip osteoarthritis: a scoping review. Osteoarthr Cartilage. 2022;30(2):260–9. 10.1016/j.joca.2021.11.008 34800632

[14] Gardner T, Refshauge K, McAuley J, Goodall S, Hübscher M, Smith L. Patient led goal setting in chronic low back pain-What goals are important to the patient and are they aligned to what we measure? Patient Educ Couns. 2015;98(8):1035–8. 25959985 10.1016/j.pec.2015.04.012

[15] Peters MDJ, Marnie C, Tricco AC, Pollock D, Munn Z, Alexander L, et al. Updated methodological guidance for the conduct of scoping reviews. JBI Evid Synth. 2020;18(10):2119. 33038124 10.11124/JBIES-20-00167

[16] Tricco AC, Lillie E, Zarin W, O’Brien KK, Colquhoun H, Levac D, et al. PRISMA extension for scoping reviews (PRISMA-ScR): checklist and explanation. Ann Intern Med. 2018;169(7):467–73. 30178033 10.7326/M18-0850

[17] Southerst D, Hincapié CA, Yu H, Verville L, Bussières A, Gross DP, et al. Systematic review to inform a world health organization (WHO) clinical practice guideline: benefits and harms of structured and standardized education or advice for chronic primary low back pain in adults. J Occup Rehabil. 2023;33(4):625–35. 37991651 10.1007/s10926-023-10120-8 PMC10684630

[18] Thematic Analysis Generator [Internet]. 2025. (galaxy.ai). Available from: https://galaxy.ai/ai-thematic-analysis-generator

[19] Oral A, Arman S, Tarakci E, Patrini M, Arienti C, Etemadi Y, et al. A systematic review of clinical practice guidelines for persons with osteoarthritis. A ‘best evidence for rehabilitation’ (be4rehab) paper to develop the WHO’s package of interventions for rehabilitation: a systematic review of clinical practice guidelines for persons with osteoarthritis for the identification of best evidence for rehabilitation. Int J Rheum Dis. 2022;25(4):383–93. 35166450 10.1111/1756-185X.14292

[20] Ernstzen DV, Hillier SL, Louw QA. Synthesis of clinical practice guideline recommendations for the primary health care of chronic musculoskeletal pain. J Eval Clin Pract. 2022;28(3):454–67. 34913219 10.1111/jep.13644

[21] Wong JJ, Côté P, Sutton DA, Randhawa K, Yu H, Varatharajan S, et al. Clinical practice guidelines for the noninvasive management of low back pain: a systematic review by the Ontario protocol for traffic injury management (OPTIMa) collaboration. Eur J Pain. 2017;21(2):201–16. 27712027 10.1002/ejp.931

[22] Gay C, Chabaud A, Guilley E, Coudeyre E. Educating patients about the benefits of physical activity and exercise for their hip and knee osteoarthritis. Systematic literature review. Ann Phys Rehabil Med. 2016;59(3):174–83. 27053003 10.1016/j.rehab.2016.02.005

[23] Nelson AE, Allen KD, Golightly YM, Goode AP, Jordan JM. A systematic review of recommendations and guidelines for the management of osteoarthritis: the chronic osteoarthritis management initiative of the U.S. bone and joint initiative. Semin Arthritis Rheum. 2014;43(6):701–12. 24387819 10.1016/j.semarthrit.2013.11.012

[24] Conley B, Bunzli S, Bullen J, O’Brien P, Persaud J, Gunatillake T, et al. Core recommendations for osteoarthritis care: a systematic review of clinical practice guidelines. Arthritis Care Res. 2023;75(9):1897–907. 10.1002/acr.25101 PMC10952362 36762545

[25] Alves GS, Vera GEZ, Maher CG, Ferreira GE, Machado GC, Buchbinder R, et al. Clinical care standards for the management of low back pain: a scoping review. Rheumatol Int. 2024;44(7):1197–207. 38421427 10.1007/s00296-024-05543-2 PMC11178557

[26] Bichsel D, Liechti FD, Schlapbach JM, Wertli MM. Cross-sectional analysis of recommendations for the treatment of hip and knee osteoarthritis in clinical guidelines. Arch Phys Med Rehabil. 2022;103(3):559-569.e5. 34411512 10.1016/j.apmr.2021.07.801

[27] Tittlemier BJ, Wittmeier KD, Webber SC. Quality and content analysis of clinical practice guidelines which include nonpharmacological interventions for knee osteoarthritis. J Eval Clin Pract. 2021;27(1):93–102. 32219960 10.1111/jep.13391

[28] Lim TH, Mak HY, Man Ngai SM, Man YT, Tang CH, Wong AYL, et al. Nonpharmacological spine pain management in clinical practice guidelines: a systematic review using AGREE II and AGREE-REX tools. J Orthop Sports Phys Ther. 2025;55(1):12–25. 39680669 10.2519/jospt.2024.12729

[29] McKenzie BJ, Haas R, Ferreira GE, Gorelik A, Cyril S, Han JX, et al. Agreement between high-quality clinical practice guidelines in their treatment recommendations for low back pain: a systematic review. Spine J Off J North Am Spine Soc. 2025;(101130732). 10.1016/j.spinee.2025.07.015 40639620

[30] Núñez-Cortés R, Salazar-Méndez J, Calatayud J, Malfliet A, Lluch E, Mendez-Rebolledo G, et al. The optimal dose of pain neuroscience education added to an exercise programme for patients with chronic spinal pain: a systematic review and dose-response meta-analysis. Pain. 2024;165(6):1196–206. 38047772 10.1097/j.pain.0000000000003126

[31] Ma X, Chen R, Li W, Huang P. A systematic review and meta-analysis of pain neuroscience education for chronic low back pain: short-term outcomes of pain and disability. Physiother Theory Pract. 2024;40(9):2130–49. 37395152 10.1080/09593985.2023.2232003

[32] Shin S, Kim H. Carryover effects of pain neuroscience education on patients with chronic lower back pain: a systematic review and meta-analysis. Med Kaunas Lith. 2023;59(7):1268. 10.3390/medicina59071268 PMC10383026 37512079

[33] Wood L, Hendrick PA. A systematic review and meta-analysis of pain neuroscience education for chronic low back pain: short-and long-term outcomes of pain and disability. Eur J Pain. 2019;23(2):234–49. 30178503 10.1002/ejp.1314

[34] Tegner H, Frederiksen P, Esbensen BA, Juhl C. Neurophysiological pain education for patients with chronic low back pain: a systematic review and meta-analysis. Clin J Pain. 2018;34(8):778–86. 29443723 10.1097/AJP.0000000000000594

[35] Puri BK, Theodoratou M. The efficacy of psychoeducation in managing low back pain: a systematic review. Psychiatr Psychiatr. 2023;34(3):231–42. 10.22365/jpsych.2022.104 36538822

[36] Furlong B, Etchegary H, Aubrey-Bassler K, Swab M, Pike A, Hall A. Patient education materials for non-specific low back pain and sciatica: a systematic review and meta-analysis. PLoS ONE. 2022;17(10):e0274527. 36223377 10.1371/journal.pone.0274527 PMC9555681

[37] Ho EKY, Chen L, Simic M, Ashton-James CE, Comachio J, Wang DXM, et al. Psychological interventions for chronic, non-specific low back pain: systematic review with network meta-analysis. BMJ. 2022;30(376):e067718. 10.1136/bmj-2021-067718 PMC8965745 35354560

[38] Gomes LA, Rodrigues AM, van der Windt D, Pires D, Afreixo V, Canhão H, et al. Minimal intervention of patient education for low back pain: a systematic review with meta-analysis. J Orthop Sports Phys Ther. 2024;54(2):107–19. 37970797 10.2519/jospt.2023.11865

[39] Jones CM, Shaheed CA, Ferreira GE, Kharel P, Christine Lin CW, Maher CG. Advice and education provide small short-term improvements in pain and disability in people with non-specific spinal pain: a systematic review. J Physiother. 2021;67(4):263–70. 34518145 10.1016/j.jphys.2021.08.014

[40] Zahari Z, Ishak A, Justine M. The effectiveness of patient education in improving pain, disability and quality of life among older people with low back pain: a systematic review. J Back Musculoskelet Rehabil. 2020;33(2):245–54. 31356191 10.3233/BMR-181305

[41] Lin LH, Lin TY, Chang KV, Wu WT, Özçakar L. Pain neuroscience education for reducing pain and kinesiophobia in patients with chronic neck pain: a systematic review and meta-analysis of randomized controlled trials. Eur J Pain. 2024;28(2):231–43. 37694895 10.1002/ejp.2182

[42] Yu H, Côté P, Southerst D, Wong JJ, Varatharajan S, Shearer HM, et al. Does structured patient education improve the recovery and clinical outcomes of patients with neck pain? A systematic review from the Ontario protocol for traffic injury management (OPTIMa) collaboration. Spine J. 2016;16(12):1524–40. 24704678 10.1016/j.spinee.2014.03.039

[43] Ordoñez-Mora LT, Morales-Osorio MA, Rosero ID. Effectiveness of interventions based on pain neuroscience education on pain and psychosocial variables for osteoarthritis: a systematic review. Int J Environ Res Public Health. 2022;19(5):2559. 35270250 10.3390/ijerph19052559 PMC8909562

[44] Lesmond I, Calvache-Mateo A, Heredia-Ciuró A, Martín-Núñez J, Navas-Otero A, López-López L, et al. Neurophysiological pain education for patients with symptomatic knee osteoarthritis: a systematic review and meta-analysis. Patient Educ Couns. 2024;120:108128. 38147773 10.1016/j.pec.2023.108128

[45] Uritani D, Koda H, Sugita S. Effects of self-management education programmes on self-efficacy for osteoarthritis of the knee: a systematic review of randomised controlled trials. BMC Musculoskelet Disord. 2021;22(1):515. 34090406 10.1186/s12891-021-04399-y PMC8180097

[46] Kroon FPB, van der Burg LRA, Buchbinder R, Osborne RH, Johnston RV, Pitt V. Self-management education programmes for osteoarthritis. Cochrane Database Syst Rev. 2014;201(1):8963. 10.1002/14651858.CD008963.pub2 PMC11104559 24425500

[47] Wang L, Zhang L, Yang L, Cheng-qi H. Effectiveness of pain coping skills training on pain, physical function, and psychological outcomes in patients with osteoarthritis: a systemic review and meta-analysis. Clin Rehabil. 2021;35(3):342–55. 33103915 10.1177/0269215520968251

[48] Sasaki R, Honda Y, Oga S, Fukushima T, Tanaka N, Kajiwara Y, et al. Effect of exercise and/or educational interventions on physical activity and pain in patients with hip/knee osteoarthritis: a systematic review with meta-analysis. PLoS ONE. 2022;17(11):e0275591. 36409668 10.1371/journal.pone.0275591 PMC9678259

[49] Sinatti P, Sánchez Romero EA, Martínez-Pozas O, Villafañe JH. Effects of patient education on pain and function and its impact on conservative treatment in elderly patients with pain related to hip and knee osteoarthritis: a systematic review. Int J Environ Res Public Health. 2022;19(10):6194. 35627729 10.3390/ijerph19106194 PMC9140798

[50] Goff AJ, De Oliveira Silva D, Merolli M, Bell EC, Crossley KM, Barton CJ. Patient education improves pain and function in people with knee osteoarthritis with better effects when combined with exercise therapy: a systematic review. J Physiother. 2021;67(3):177–89. 34158270 10.1016/j.jphys.2021.06.011

[51] Bülow K, Lindberg K, Vaegter HB, Juhl CB. Effectiveness of pain neurophysiology education on musculoskeletal pain: a systematic review and meta-analysis. Pain Med Malden Mass. 2021;22(4):891–904. 10.1093/pm/pnaa484 33764394

[52] Siddall B, Ram A, Jones MD, Booth J, Perriman D, Summers SJ. Short-term impact of combining pain neuroscience education with exercise for chronic musculoskeletal pain: a systematic review and meta-analysis. Pain. 2022;163(1):e20-30. 33863860 10.1097/j.pain.0000000000002308

[53] Salazar-Méndez J, Leão Ribeiro I, Garrido-Castillo M, Gacitúa J. Effects of pain neuroscience education on psycho-emotional and cognitive variables in individuals with chronic musculoskeletal pain: a systematic review of randomised clinical trials. Eur J Physiother. 2024;26(1):33–41.

[54] Salazar-Méndez J, Núñez-Cortés R, Suso-Martí L, Ribeiro IL, Garrido-Castillo M, Gacitúa J, et al. Dosage matters: uncovering the optimal duration of pain neuroscience education to improve psychosocial variables in chronic musculoskeletal pain. A systematic review and meta-analysis with moderator analysis. Neurosci Biobehav Rev. 2023;153:105328. 37516218 10.1016/j.neubiorev.2023.105328

[55] Lepri B, Romani D, Storari L, Barbari V. Effectiveness of pain neuroscience education in patients with chronic musculoskeletal pain and central sensitization: a systematic review. Int J Environ Res Public Health. 2023;20(5):4098. 36901108 10.3390/ijerph20054098 PMC10001851

[56] Louw A, Zimney K, Puentedura EJ, Diener I. The efficacy of pain neuroscience education on musculoskeletal pain: a systematic review of the literature. Physiother Theory Pract. 2016;32(5):332–55. 27351541 10.1080/09593985.2016.1194646

[57] Romm MJ, Ahn S, Fiebert I, Cahalin LP. A meta-analysis of therapeutic pain neuroscience education, using dosage and treatment format as moderator variables. Pain Pract. 2021;21(3):366–80. 33131210 10.1111/papr.12962

[58] Watson JA, Ryan CG, Cooper L, Ellington D, Whittle R, Lavender M, et al. Pain neuroscience education for adults with chronic musculoskeletal pain: a mixed-methods systematic review and meta-analysis. J Pain. 2019;20(10):1140.e1-1140.e22. 30831273 10.1016/j.jpain.2019.02.011

[59] Mullins CF, Bak B, Moore D. Pre-outpatient group education and assessment in chronic pain: a systematic review. Pain Med Malden Mass. 2022;23(1):89–104. 10.1093/pm/pnab036 33787896

[60] Salwana Kamsan S, Kaur Ajit Singh D, Pin Tan M, Kumar S. Systematic review on the contents and parameters of self-management education programs in older adults with knee osteoarthritis. Australas J Ageing. 2021;40(1):e1-12. 32881241 10.1111/ajag.12844

[61] Isaji Y, Kurasawa Y, Sasaki D, Hayashi M, Kitagawa T. Psychological intervention for knee osteoarthritis: a systematic review and meta-analysis. Psychol Health Med. 2025;30(3):636–62. 39873210 10.1080/13548506.2025.2454039

[62] Migliorini F, Maffulli N, Schafer L, Manocchio N, Bossa M, Foti C, et al. Impact of education in patients undergoing physiotherapy for lower back pain: a level I systematic review and meta-analysis. Eur J Trauma Emerg Surg. 2025;51(1):113. 39969656 10.1007/s00068-025-02788-9 PMC11839871

[63] Nunez-Cortes R, Salazar-Mendez J, Calatayud J, Lluch E, Lopez-Bueno R, Horment-Lara G, et al. How do the target concepts of pain science education combined with exercise contribute to the effect on pain intensity and disability in patients with chronic spinal pain? A systematic review and meta-analysis with moderator analysis. Neurosci Biobehav Rev. 2024;163(Alyousef, B., Kazemi, Z., Cicuttini, F.M., Heritier, S., Wang, Y., Urquhart, D.M. (2023). High levels of back disability, not back pain, are associated with reduced physical activity across key activity domains. Musculoskelet. Sci. Pract. 65. https://pu). 10.1016/j.msksp.2023.102768 37126982

[64] Palahi-Calsina I, Jubany J, Sordo L, Lorente S, Espelt A, Borao O. Effectiveness of pain neuroscience education among adults with chronic neck pain. Systematic review. . Eur J Physiother. 2025;27(3):147–58.

[65] Piano L, Audasso P, Benzi L, Occhionero A, Trucco M, Innocenti T, et al. Individual education for patients with chronic low back pain: likely a clinically relevant effect for long-term disability compared to noneducational interventions. A systematic review with meta-analysis. J Orthop Sports Phys Ther. 2025;55(5):331–43. 40266700 10.2519/jospt.2025.12794

[66] Sanchez-Robalino A, Sinchi-Sinchi H, Ramirez A. Effectiveness of pain neuroscience education in physical therapy: a systematic review and meta-analysis. Brain Sci. 2025. 10.3390/brainsci15060658. 40563828 10.3390/brainsci15060658 PMC12191368

[67] Simick Behera N, Duong V, Eyles J, Cui H, Gould D, Barton C, et al. How does osteoarthritis education influence knowledge, beliefs, and behavior in people with knee and hip osteoarthritis? A systematic review. Arthritis Care Res. 2024;76(11):1511–31. 10.1002/acr.25391 38923866

[68] Tatikola SP, Natarajan V, Amaravadi SK, Desai VK, Asirvatham AR, Nagaraja R. Effect of pain neuroscience education+ (PNE+) in people with different mechanisms of chronic pain: a systematic review and meta-analysis. J Bodyw Mov Ther. 2025;41(9700068):215–37. 39663091 10.1016/j.jbmt.2024.11.016

[69] Thompson K, Johnson MI, Milligan J, Briggs M. Rethinking pain education from the perspectives of people experiencing pain: a meta-ethnography to inform physiotherapy training. BMJ Open. 2022;12(1):e046363. 35017228 10.1136/bmjopen-2020-046363 PMC8753399

[70] Lim YZ, Chou L, Au RT, Seneviwickrama KMD, Cicuttini FM, Briggs AM, et al. People with low back pain want clear, consistent and personalised information on prognosis, treatment options and self-management strategies: a systematic review. J Physiother. 2019;65(3):124–35. 31227280 10.1016/j.jphys.2019.05.010

[71] Ciolan F, Bertoni G, Crestani M, Falsiroli Maistrello L, Coppola I, Rossettini G, et al. Perceived factors influencing the success of pain neuroscience education in chronic musculoskeletal pain: a meta-synthesis of qualitative studies. Disabil Rehabil. 2025;47(10):2459–74. 39225055 10.1080/09638288.2024.2398141

[72] Cuenca-Martínez F, Suso-Martí L, Calatayud J, Ferrer-Sargues FJ, Muñoz-Alarcos V, Alba-Quesada P, et al. Pain neuroscience education in patients with chronic musculoskeletal pain: an umbrella review. Front Neurosci. 2023;17:1272068. 38075271 10.3389/fnins.2023.1272068 PMC10704151

[73] Martinez-Calderon J, Ho EKY, Ferreira PH, Garcia-Muñoz C, Villar-Alises O, Matias-Soto J. A call for improving research on pain neuroscience education and chronic pain: an overview of systematic reviews. J Orthop Sports Phys Ther. 2023;53(6):353–68. 37161889 10.2519/jospt.2023.11833

[74] Adenis N, Gosselin K, Stetsenko N, Thevenon A. Clarification of the ‘pain neuroscience education’ concept in the management of patients with persistent low back pain: a scoping review. J Back Musculoskelet Rehabil. 2023;36(5):995–1010. 37458022 10.3233/BMR-220370

[75] Chou L, Ranger TA, Peiris W, Cicuttini FM, Urquhart DM, Sullivan K, et al. Patients’ perceived needs of health care providers for low back pain management: a systematic scoping review [b]. Spine J Off J North Am Spine Soc. 2018;18(4):691–711. 10.1016/j.spinee.2018.01.006 29373836

[76] Opara M, Kozinc Ž. Pain neuroscience education in management of non-traumatic neck pain: a scoping review. Eur J Physiother. 2024;26(6):326–36.

[77] Salazar-Méndez J, Cuyul-Vásquez I, Ponce-Fuentes F, Guzmán-Muñoz E, Núñez-Cortés R, Huysmans E, et al. Pain neuroscience education for patients with chronic pain: a scoping review from teaching-learning strategies, educational level, and cultural perspective. Patient Educ Couns. 2024;123:108201. 38387389 10.1016/j.pec.2024.108201

[78] Debonne C, Houdart A, Cachinho C, Ouvrier-Neyret A, Gerard T, Vaillant V, et al. Accessible patient education materials for low back pain rarely meet people’s information needs: a scoping review. Musculoskelet Care. 2025;23(2):e70130. 10.1002/msc.70130 PMC12124170 40444958

[79] Watson JA, Ryan CG, Atkinson G, Williamson P, Ellington D, Whittle R, et al. Inter-individual differences in the responses to pain neuroscience education in adults with chronic musculoskeletal pain: a systematic review and meta-analysis of randomized controlled trials. J Pain. 2021;22(1):9–20. 32585363 10.1016/j.jpain.2020.03.006

[80] Goff AJ, de Oliveira SD, Ezzat AM, Bell EC, Crossley KM, O’Halloran P, et al. Knee osteoarthritis education interventions in published trials are typically unclear, not comprehensive enough, and lack robust development: ancillary analysis of a systematic review. J Orthop Sports Phys Ther. 2022;52(5):276–86. 34905960 10.2519/jospt.2022.10771

[81] French SD, Bennell KL, Nicolson PJA, Hodges PW, Dobson FL, Hinman RS. What do people with knee or hip osteoarthritis need to know? An international consensus list of essential statements for osteoarthritis. Arthritis Care Res. 2015;67(6):809–16. 10.1002/acr.22518 25418120

[82] French SD, Nielsen M, Hall L, Nicolson PJA, van Tulder M, Bennell KL, et al. Essential key messages about diagnosis, imaging, and self-care for people with low back pain: a modified Delphi study of consumer and expert opinions. Pain. 2019;160(12):2787–97. 31356451 10.1097/j.pain.0000000000001663

[83] Palahi-Calsina I, Jubany J, Sordo L, Lorente S, Espelt A, Borao O. Effectiveness of pain neuroscience education among adults with chronic neck pain. Systematic review. Eur J Physiother. 2025;27(3):147EP – 158.

[84] Núñez-Cortés R, Salazar-Méndez J, Calatayud J, Lluch E, López-Bueno R, Horment-Lara G, et al. How do the target concepts of pain science education combined with exercise contribute to the effect on pain intensity and disability in patients with chronic spinal pain? A systematic review and meta-analysis with moderator analysis. Neurosci Biobehav Rev. 2024;1(163):105740. 10.1016/j.neubiorev.2024.105740 38852291

[85] Mullins CF, Bak B, Moore D. Pre-outpatient group education and assessment in chronic pain: a systematic review. Pain Med. 2022;23(1):89–104. 33787896 10.1093/pm/pnab036

[86] Hartvigsen J, Hancock MJ, Kongsted A, Louw Q, Ferreira ML, Genevay S, et al. What low back pain is and why we need to pay attention. Lancet. 2018;391(10137):2356–67. 29573870 10.1016/S0140-6736(18)30480-X

[87] Hoole S, Bell P, Conaghan PG, Cottrel E, Halstead-Rastrick J, Inglesfield J, et al. Osteoarthritis in over 16s: diagnosis and management [Internet]. London: National Institute for Health and Care Excellence (NICE); 2022 [cited 2025 Mar 24]. (National Institute for Health and Care Excellence: Guidelines). Available from: http://www.ncbi.nlm.nih.gov/books/NBK588843/

[88] Grøn S, Johansson M, Schiphof D, Koes B, Kongsted A. Do self-management supportive interventions reduce healthcare utilization for people with musculoskeletal pain conditions?: a systematic review. Public Health. 2025;238:152–61. 39662130 10.1016/j.puhe.2024.10.021

[89] Jonkman NH, Schuurmans MJ, Jaarsma T, Shortridge-Baggett LM, Hoes AW, Trappenburg JC. Self-management interventions: proposal and validation of a new operational definition. J Clin Epidemiol. 2016;80:34–42. 27531245 10.1016/j.jclinepi.2016.08.001

[90] Marques MM, Wright AJ, Corker E, Johnston M, West R, Hastings J, et al. The behaviour change technique ontology: transforming the behaviour change technique taxonomy v1. Wellcome Open Res. 2023;8:308. 37593567 10.12688/wellcomeopenres.19363.2 PMC10427801

[91] Wright AJ, Zhang L, Howes E, Veall C, Corker E, Johnston M, et al. Specifying how intervention content is communicated: development of a style of delivery ontology. Wellcome Open Res. 2023;8:456. 39193088 10.12688/wellcomeopenres.19899.1 PMC11347912

