# Supplementary material for: Patient education for chronic musculoskeletal pain: a scoping review of recommendations, effectiveness, and educational content
Source: Chiropr Man Therap. 2026 Jan 2;34:3. doi: 10.1186/s12998-025-00614-y (PMC12865995; doi:10.1186/s12998-025-00614-y)
Supplement: Supplementary file 1 — Supplementary Material 1 [file 12998_2025_614_MOESM1_ESM.docx]

Supplementary 1. Search Strategy

1. **Database & platform:** MEDLINE (Ovid)

**Years of search:** 2014-2024

**Data search run:** March 15, 2024 updated July 14, 2025

**Numbers of records retrieved:** 1.564 (update 3.380)

Search strategy:

| \| 1. back pain/ or low back pain/ \| \| --- \| \| 2. spinal injuries/ \| \| 3. exp spondylitis/ \| \| 4. exp osteoarthritis \| \| 5. exp sciatic neuropathy \| \| 6. intervertebral disc degeneration/ \| \| 7. intervertebral disc displacement/ \| \| 8. spinal stenosis/ \| \| 9. exp spondylosis \| \| 10. fibromyalgia/ \| \| 11. musculoskeletal pain/ \| \| 12. myalgia/ \| \| 13. myofascial pain syndrome/ \| \| 14. exp tendon injuries/ \| \| 15. chronic pain/ \| \| 16. exp arthralgia/ \| \| 17. exp neck pain/ \| \| 18. exp pelvic pain \| \| 19. exp neck injuries/ \| \| 20. exp back injuries/ \| \| 21. radiculopathy/ \| \| 22. patellofemoral pain syndrome/  23. shoulder impingement syndrome/   \| 24. ((low* adj2 (back adj2 pain*)) or (low-back* adj2 pain*) or (lower-back* adj2 pain*) or (low* adj2 back- pain*)).ti,ab,kf \| \| --- \| \| 25. ((low* adj2 (back adj2 injur*)) or (low-back* adj2 injur*) or (lower-back* adj2 injur*) or (low* adj2 back-injur*)).ti,ab,kf \| \| 26. ((low* adj2 (back adj2 trauma*)) or (low-back adj2 trauma*) or (lower-back* adj2 trauma*) or (low* adj2 back-trauma*)).ti,ab,kf \| \| 27. (lumb?* adj3 (dis?* adj3 (extru* or degenerat* or displac* or herniat* or prolaps* or sequestered or slipped or protru* or avuls*))).ti,ab,kf \| \| 28. (lumb?* adj3 (pain* or facet* or (nerve adj2 root*) or osteoarth* or radicul* or stenos* or spondylo* or zygapophys* or injur* or discomfort* or dysfunction* or sore* or herniat*)).ti,ab,kf \| \| 29. (back adj3 (ach* or injur* or pain* or sprain* or strain* or disorder* or mechanical or syndrome* or ach*)).ti,ab,kf \| \| 30. (intervertebral* adj3 (dis?* adj3 (extru* or degenerat* or displac* or herniat* or prolaps* or sequestered or slipped or protru* or avuls*))).ti,ab,kf \| \| 31. (coccy* adj2 (ach* or injur* or pain* or sprain* or strain*)).ti,ab,kf \| \| 32. (coccygodyn* or coccalg* or coccygalg*).ti,ab,kf \| \| 33. (lumbago* adj3 (pain* or symptom* or disabilit* or radiat* or leg* or spine* or spinal*)).ti,ab,kf \| \| 34. lumboischialg*.ti,ab,kf \| \| 35. (piriformis* adj2 syndrome*).ti,ab,kf \| \| 36. (sacr?* adj3 (pain* or facet* or (nerve adj2 root*) or osteoarth* or radicul* or stenos* or spondylo* or zygapophys* or injur* or discomfort* or dysfunction* or sore* or herniat*)).ti,ab,kf \| \| 37. (sacr?* adj2 (ach* or injur* or pain* or sprain* or strain*)).ti,ab,kf \| \| 38. ("si" adj2 (joint* adj3 (pain* or facet* or (nerve adj2 root*) or osteoarth* or radicul* or stenos* or spondylo* or zygapophys* or injur* or discomfort* or dysfunction* or sore* or herniat*))).ti,ab,kf \| \| 39. (cervical* adj3 (dis?* adj3 (extru* or degenerat* or displac* or herniat* or prolaps* or sequestered or slipped or protru* or avuls*))).ti,ab,kf \| \| 40. (cervical* adj3 (pain* or facet* or (nerve adj2 root*) or osteoarth* or radicul* or stenos* or spondylo* or zygapophys* or injur* or discomfort* or dysfunction* or sore* or herniat*)).ti,ab,kf \| \| 41. (thoracic* adj3 (dis?* adj3 (extru* or degenerat* or displac* or herniat* or prolaps* or sequestered or slipped or protru* or avuls*))).ti,ab,kf \| \| 42. (thoracic* adj3 (pain* or facet* or (nerve adj2 root*) or osteoarth* or radicul* or stenos* or spondylo* or zygapophys* or injur* or discomfort* or dysfunction* or sore* or herniat*)).ti,ab,kf \| \| 43. (sciatic* adj3 (pain* or symptom* or disabilit* or radiat* or leg* or spine* or spinal*)).ti,ab,kf \| \| 44. (stenos* adj2 (spine* or spinal* or vertebral*)).ti,ab,kf \| \| 45. ((spine* or spinal*) adj2 osteoarthr*).ti,ab,kf \| \| 46. (spinal* adj3 (condition* or diseas* or disabilit* or disorder* or degenerat* or pain* or stenos*)).ti,ab,kf \| \| 47. (spine* adj3 (condition* or diseas* or disabilit* or disorder* or degenerat* or pain* or stenos*)).ti,ab,kf \| \| 48. (spondyl* adj3 (condition* or diseas* or disabilit* or disorder* or degenerat* or pain*)).ti,ab,kf \| \| 49. (radiculopath* adj3 (lumbar* or lumbo* or sacral* or sacro* or low-back* or lower-back* or cervical* or thoracic or spine* or spinal*)).ti,ab,kf \| \| 50. (radiating* adj3 (lumbar* or lumbo* or sacral* or sacro* or low-back* or lower-back* or cervical* or thoracic or spine* or spinal*)).ti,ab,kf \| \| 51. (radicular* adj3 (lumbar* or lumbo* or sacral* or sacro* or low-back* or lower-back* or cervical* or thoracic or spine* or spinal*)).ti,ab,kf \| \| 52. (osteoarth* adj2 (knee* or hip*)).ti,ab,kf \| \| 53. (musculoskeletal adj2 (pain* or disorder* or syndrome*)).ti,ab,kf \| \| 54. (fibromyalgia adj3 (pain* or disorder* or disabilit* or diseas* or musculoskeletal* or symptom* or chronic)).ti,ab,kf \| \| 55. (myofascial adj2 (pain* or syndrome*)).ti,ab,kf \| \| 56. (tendinopath* adj2 (knee* or hip* or shoulder*)).ti,ab,kf \| \| 57. (tendinit* adj2 (knee* or hips* or shoulder*)).ti,ab,kf \| \| 58. (neck adj2 (pain* or injur* or syndrome* or disorder*)).ti,ab,kf \| \| 59. shoulder* adj2 (pain* or injur*).ti,ab,kf \| \| 60. knee* adj2 (pain* or injur* or syndrome* or disorder*).ti,ab,kf \| \| 61. hip* adj2 (pain* or injur* or syndrome* or disorder*).ti,ab,kf \| \| 62. patellofemoral adj2 (pain* or syndrome* or disorder*).ti,ab,kf \| \| 63. shoulder adj2 (impingement* or syndrome* or disorder*).ti,ab,kf \| \| 64. (rotator cuff* adj2 (syndrome* or diseas* or injur* or tear* or damage*)).ti,ab,kf \| \| 65. persistent adj3 (pain* or syndrome*).ti,ab,kf \| \| 66. pain adj2 (disorder* or syndrome* or musculoskeletal* or myofascial).ti,ab,kf \| \| 67. (chronic adj2 (pain* or syndrome* or musculoskeletal)).ti,ab,kf \| \| 68. longstanding pain*.ti,ab,kf \| \| 69. (whiplash* adj2 (pain* or syndrome* or associated or disorder*)).ti,ab,kf  70. or/1-69   \| 71. patient education as Topic/ \| \| --- \| \| 72. exp consumer health information/ \| \| 73. self care/ \| \| 74. patient participation/ \| \| 75. patient compliance/ \| \| 76. self-management/ \| \| 77. education/ \| \| 78. health promotion/ \| \| 79. patient education handout/   \| 80. (patient adj2 (education* or advice or reassur*)).ti,ab,kf \| \| --- \| \| 81. consumer adj2 health information*.ti,ab,kf \| \| 82. (self* adj2 (care* or self-care*)).ti,ab,kf \| \| 83. ((self* adj2 manag*) or self-manag*).ti,ab,kf \| \| 84. ((self* adj help*) or self-help*).ti,ab,kf \| \| 85. patient adj2 (empowerment*).ti,ab,kf \| \| 86. (health adj2 (information* or literacy* or promotion)).ti,ab,kf \| \| 87. (pain adj2 (education* or coping* or management*)).ti,ab,kf \| \| 88. (therapeutic adj2 education).ti,ab,kf \| \| 89. (explain adj2 pain).ti,ab,kf \| \| 90. psychoeducation*.ti,ab,kf \| \| 91. ((patient education*) adj4 (online* or written* or oral* or verbal* or digital* or health*)).ti,ab,kf \| \| 92. (education adj2 (neuroscience* or neurobiolog* or neurophysiolog*)).ti,ab,kf \| \| 93. (patient education* adj4 (app or apps or application* or internet or website* or web-site* or web-base* or (web* adj2 page*) or (web* adj2 application*) or (web* adj2 interfac*))).ti,ab,kf \| \| 94. (behavio?ral adj2 (education* or intervention*)).ti,ab,kf \| \| 95. reassuring adj2 information*.ti,ab,kf \| \| 96. (ergonomic* adj2 (education* or advice*)).ti,ab,kf \| \| 97. (patient education* adj3 (individual or group* or group-based or face-to-face)).ti,ab,kf  98. or/71-97   \| 99. systematic review/ \| \| --- \| \| 100. meta-analysis/ \| \| 101. exp "review literature as topic"/ \| \| 102. exp meta-analysis as topic \| \| 103. delphi technique/ \| \| 104. exp consensus development conference/ \| \| 105. guideline/   \| 106. systematic* adj2 (review* or overview).ti,ab,kf \| \| --- \| \| 107. meta?anal*.ti,ab,kf \| \| 108. network meta?anal*.ti,ab,kf \| \| 109. network meta anal*.ti,ab,kf \| \| 110. (consensus adj2 (report* or statement* or development*)).ab,ti,kf \| \| 111. (delphi adj2 (stud* or technique* or process* or consensus)).ti,ab,kf \| \| 112. (guideline* adj (international or national)).ti,ab,kf \| \| 113. or/ 99-112  114. 70 AND 98 AND 113  limit 114 to yr="2014 - 2024" \|  \|  \| \| --- \| \|  \| \|  \| \|  \| \|  \| \|  \| \|  \| \|  \| \|  \| \| \| \| \| \| \|  \| |
| --- | --- | --- | --- | --- | --- | --- | --- | --- | --- | --- | --- | --- | --- | --- | --- | --- | --- | --- | --- | --- | --- | --- | --- | --- | --- | --- | --- | --- | --- | --- | --- | --- | --- | --- | --- | --- | --- | --- | --- | --- | --- | --- | --- | --- | --- | --- | --- | --- | --- | --- | --- | --- | --- | --- | --- | --- | --- | --- | --- | --- | --- | --- | --- | --- | --- | --- | --- | --- | --- | --- | --- | --- | --- | --- | --- | --- | --- | --- | --- | --- | --- | --- | --- | --- | --- | --- | --- | --- | --- | --- | --- | --- | --- | --- | --- | --- | --- | --- | --- | --- | --- | --- | --- | --- | --- | --- | --- | --- | --- | --- | --- | --- | --- | --- | --- | --- | --- | --- | --- | --- |

1. **Database & platform:** EMBASE (Ovid)

**Years of search:** 2014 - 2024

**Data search run:** March 15, 2024 updated July 14, 2025

**Numbers of records retrieved:** 2.573 (update 6.069)

Search strategy:

| \| 1. exp backache/ \| \| --- \| \| 2. intervertebral disk disease/ or spinal pain/ or spine injury/ or spine instability/ or spine malformation/ or spondylitis/ or spondylolisthesis/ or spondylolysis/ or spondylosis/ or vertebral canal stenosis/ \| \| 3. exp spinal osteoarthritis/ \| \| 4. exp osteoarthritis/ \| \| 5. exp sciatica/ \| \| 6. exp ischialgia/ \| \| 7. neck injury/ \| \| 8. exp whiplash injury/ \| \| 9. exp radiculopathy/ \| \| 10. exp tendinitis/ \| \| 11. exp complex regional pain syndrome/ \| \| 12. chronic pain/ \| \| 13. exp patellofemoral pain syndrome/ \| \| 14. exp hip pain \| \| 15. exp knee pain/ \| \| 16. exp arthralgia/ \| \| 17. exp pelvic pain/ \| \| 18. musculoskeletal injury/   \| 19. ((low* adj2 (back adj2 pain*)) or (low-back* adj2 pain*) or (lower-back* adj2 pain*) or (low* adj2 back-pain*)).ti,ab,kf \| \| --- \| \| 20. ((low* adj2 (back adj2 injur*)) or (low-back* adj2 injur*) or (lower-back* adj2 injur*) or (low* adj2 back-injur*)).ti,ab,kf \| \| 21. ((low* adj2 (back adj2 trauma*)) or (low-back adj2 trauma*) or (lower-back* adj2 trauma*) or (low* adj2 back-trauma*)).ti,ab,kf \| \| 22. (lumb?* adj3 (dis?* adj3 (extru* or degenerat* or displac* or herniat* or prolaps* or sequestered or slipped or protru* or avuls*))).ti,ab,kf \| \| 23. (lumb?* adj3 (pain* or facet* or (nerve adj2 root*) or osteoarth* or radicul* or stenos* or spondylo* or zygapophys* or injur* or discomfort* or dysfunction* or sore* or herniat*)).ti,ab,kf \| \| 24. (back adj3 (ach* or injur* or pain* or sprain* or strain* or disorder* or mechanical or syndrome* or ach*)).ti,ab,kf \| \| 25. (intervertebral* adj3 (dis?* adj3 (extru* or degenerat* or displac* or herniat* or prolaps* or sequestered or slipped or protru* or avuls*))).ti,ab,kf \| \| 26. (coccy* adj2 (ach* or injur* or pain* or sprain* or strain*)).ti,ab,kf \| \| (coccygodyn* or coccalg* or coccygalg*).ti,ab,kf \| \| 27. (lumbago* adj3 (pain* or symptom* or disabilit* or radiat* or leg* or spine* or spinal*)).ti,ab,kf \| \| 28. lumboischialg*.ti,ab,kf \| \| 29. (piriformis* adj2 syndrome*).ti,ab,kf \| \| 30. (sacr?* adj3 (pain* or facet* or (nerve adj2 root*) or osteoarth* or radicul* or stenos* or spondylo* or zygapophys* or injur* or discomfort* or dysfunction* or sore* or herniat*)).ti,ab,kf \| \| 31. (sacr?* adj2 (ach* or injur* or pain* or sprain* or strain*)).ti,ab,kf \| \| 32. ("si" adj2 (joint* adj3 (pain* or facet* or (nerve adj2 root*) or osteoarth* or radicul* or stenos* or spondylo* or zygapophys* or injur* or discomfort* or dysfunction* or sore* or herniat*))).ti,ab,kf \| \| 33. (cervical* adj3 (dis?* adj3 (extru* or degenerat* or displac* or herniat* or prolaps* or sequestered or slipped or protru* or avuls*))).ti,ab,kf \| \| 34. (cervical* adj3 (pain* or facet* or (nerve adj2 root*) or osteoarth* or radicul* or stenos* or spondylo* or zygapophys* or injur* or discomfort* or dysfunction* or sore* or herniat*)).ti,ab,kf \| \| 35. (thoracic* adj3 (dis?* adj3 (extru* or degenerat* or displac* or herniat* or prolaps* or sequestered or slipped or protru* or avuls*))).ti,ab,kf \| \| 36. (thoracic* adj3 (pain* or facet* or (nerve adj2 root*) or osteoarth* or radicul* or stenos* or spondylo* or zygapophys* or injur* or discomfort* or dysfunction* or sore* or herniat*)).ti,ab,kf \| \| 37. (sciatic* adj3 (pain* or symptom* or disabilit* or radiat* or leg* or spine* or spinal*)).ti,ab,kf \| \| 38. (stenos* adj2 (spine* or spinal* or vertebral*)).ti,ab,kf \| \| 39. ((spine* or spinal*) adj2 osteoarthr*).ti,ab,kf \| \| 40. (spinal* adj3 (condition* or diseas* or disabilit* or disorder* or degenerat* or pain* or stenos*)).ti,ab,kf \| \| 41. (spine* adj3 (condition* or diseas* or disabilit* or disorder* or degenerat* or pain* or stenos*)).ti,ab,kf \| \| 42. (spondyl* adj3 (condition* or diseas* or disabilit* or disorder* or degenerat* or pain*)).ti,ab,kf \| \| 43. (radiculopath* adj3 (lumbar* or lumbo* or sacral* or sacro* or low-back* or lower-back* or cervical* or 44. thoracic or spine* or spinal*)).ti,ab,kf \| \| 45. (radiating* adj3 (lumbar* or lumbo* or sacral* or sacro* or low-back* or lower-back* or cervical* or thoracic or spine* or spinal*)).ti,ab,kf \| \| 46. (radicular* adj3 (lumbar* or lumbo* or sacral* or sacro* or low-back* or lower-back* or cervical* or thoracic or spine* or spinal*)).ti,ab,kf \| \| 47. (osteoarth* adj2 (knee* or hip*)).ti,ab,kf \| \| 48. (musculoskeletal adj2 (pain* or disorder* or syndrome*)).ti,ab,kf \| \| 49. (fibromyalgia adj3 (pain* or disorder* or disabilit* or diseas* or musculoskeletal* or symptom* or chronic)).ti,ab,kf \| \| 50. (myofascial adj2 (pain* or syndrome*)).ti,ab,kf \| \| 51. (tendinopath* adj2 (knee* or hip* or shoulder*)).ti,ab,kf \| \| 52. (tendinit* adj2 (knee* or hips* or shoulder*)).ti,ab,kf \| \| 53. (neck adj2 (pain* or injur* or syndrome* or disorder*)).ti,ab,kf \| \| 54. shoulder* adj2 (pain* or injur*).ti,ab,kf \| \| 55. knee* adj2 (pain* or injur* or syndrome* or disorder*).ti,ab,kf \| \| 56. hip* adj2 (pain* or injur* or syndrome* or disorder*).ti,ab,kf \| \| 57. patellofemoral adj2 (pain* or syndrome* or disorder*).ti,ab,kf \| \| 58. shoulder adj2 (impingement* or syndrome* or disorder*).ti,ab,kf \| \| 59. (rotator cuff* adj2 (syndrome* or diseas* or injur* or tear* or damage*)).ti,ab,kf \| \| 60. persistent adj3 (pain* or syndrome*).ti,ab,kf \| \| 61- pain adj2 (disorder* or syndrome* or musculoskeletal* or myofascial).ti,ab,kf \| \| 62. (chronic adj2 (pain* or syndrome* or musculoskeletal)).ti,ab,kf \| \| 63. longstanding pain*.ti,ab,kf \| \| 64. (whiplash* adj2 (pain* or syndrome* or associated or disorder*)).ti,ab,kf  65. or/ 1-64   \| 66. exp patient education/ \| \| --- \| \| 67. health education/ \| \| 68. exp health literacy/ \| \| 69. exp consumer health information/ \| \| 70. information/ \| \| 71. patient information/ \| \| 72. exp psychoeducation/ \| \| 73. exp self care/ \| \| 74. exp reassurance/ \| \| 75. exp empowerment/ \| \| 76. exp patient empowerment/   \| 77. (patient adj2 (education* or advice or reassur*)).ti,ab,kf \| \| --- \| \| 78. consumer adj2 health information*.ti,ab,kf \| \| 79. (self* adj2 (care* or self-care*)).ti,ab,kf \| \| 80. ((self* adj2 manag*) or self-manag*).ti,ab,kf \| \| 81. ((self* adj help*) or self-help*).ti,ab,kf \| \| 82. patient adj2 (empowerment*).ti,ab,kf \| \| 83. (health adj2 (information* or literacy* or promotion)).ti,ab,kf \| \| 84. (pain adj2 (education* or coping* or management*)).ti,ab,kf \| \| 85. (therapeutic adj2 education).ti,ab,kf \| \| 86. (explain adj2 pain).ti,ab,kf \| \| 87. psychoeducation*.ti,ab,kf \| \| 88. ((patient education*) adj4 (online* or written* or oral* or verbal* or digital* or health*)).ti,ab,kf \| \| 89. (education adj2 (neuroscience* or neurobiolog* or neurophysiolog*)).ti,ab,kf \| \| 90. (patient education* adj4 (app or apps or application* or internet or website* or web-site* or web-base* or (web* adj2 page*) or (web* adj2 application*) or (web* adj2 interfac*))).ti,ab,kf \| \| 91. (behavio?ral adj2 (education* or intervention*)).ti,ab,kf \| \| 92. reassuring adj2 information*.ti,ab,kf \| \| 93. (ergonomic* adj2 (education* or advice*)).ti,ab,kf \| \| 94. (patient education* adj3 (individual or group* or group-based or face-to-face)).ti,ab,kf  95. or/ 66-94   \| 96. "systematic review"/ \| \| --- \| \| 97. exp meta analysis/ \| \| 98. exp delphi study/ \| \| 99. exp consensus development/ \| \| 100. network analysis/ \| \| 101. guideline/   \| 102. systematic* adj2 (review* or overview).ti,ab,kf \| \| --- \| \| 103. meta?anal*.ti,ab,kf \| \| 104. network meta?anal*.ti,ab,kf \| \| 105. network meta anal*.ti,ab,kf \| \| 106. (consensus adj2 (report* or statement)).ti,ab,kf \| \| 107. (delphi adj2 (stud* or technique* or process* or consensus)).ti,ab,kf \| \| 108. (guideline* adj (international or national)).ti,ab,kf  109. or/ 96-108  110. 65 AND 95 AND 109  limit 110 to yr="2014 - 2024 \| \| \| \| \| \| |
| --- | --- | --- | --- | --- | --- | --- | --- | --- | --- | --- | --- | --- | --- | --- | --- | --- | --- | --- | --- | --- | --- | --- | --- | --- | --- | --- | --- | --- | --- | --- | --- | --- | --- | --- | --- | --- | --- | --- | --- | --- | --- | --- | --- | --- | --- | --- | --- | --- | --- | --- | --- | --- | --- | --- | --- | --- | --- | --- | --- | --- | --- | --- | --- | --- | --- | --- | --- | --- | --- | --- | --- | --- | --- | --- | --- | --- | --- | --- | --- | --- | --- | --- | --- | --- | --- | --- | --- | --- | --- | --- | --- | --- | --- | --- | --- | --- | --- | --- | --- | --- | --- | --- | --- | --- | --- | --- |
|  |

**Database & platform:** APA PsychINFO (Ovid)

**Years of search:** 2014 - 2024

**Data search run:** March 15, 2024 updated July 14, 2025

**Numbers of records retrieved:** 293 (update 647)

Search strategy:

| \| 1. back pain/ \| \| --- \| \| 2. chronic pain/ \| \| 3. myofascial pain/ \| \| 4. somatoform pain disorder/ \| \| 5. musculoskeletal disorders/ \| \| 6. musculoskeletal system/ \| \| 7. whiplash/ \| \| 8. spinal column/ \| \| 9. exp arthritis/ \| \| 10. tendons/ \| \| 11. knee/ \| \| 12. neuropathic pain/ \| \| 13. fibromyalgia/ \| \| 14. hips/   \| 15. ((low* adj2 (back adj2 pain*)) or (low-back* adj2 pain*) or (lower-back* adj2 pain*) or (low* adj2 back-pain*)).ti,ab \| \| --- \| \| 16. ((low* adj2 (back adj2 injur*)) or (low-back* adj2 injur*) or (lower-back* adj2 injur*) or (low* adj2 back-injur*)).ti,ab \| \| 17. ((low* adj2 (back adj2 trauma*)) or (low-back adj2 trauma*) or (lower-back* adj2 trauma*) or (low* adj2 back-trauma*)).ti,ab \| \| 18. (lumb?* adj3 (dis?* adj3 (extru* or degenerat* or displac* or herniat* or prolaps* or sequestered or slipped or protru* or avuls*))).ti,ab \| \| 19. (lumb?* adj3 (pain* or facet* or (nerve adj2 root*) or osteoarth* or radicul* or stenos* or spondylo* or zygapophys* or injur* or discomfort* or dysfunction* or sore* or herniat*)).ti,ab \| \| 20. (back adj3 (ach* or injur* or pain* or sprain* or strain* or disorder* or mechanical or syndrome* or ach*)).ti,ab \| \| 21. (intervertebral* adj3 (dis?* adj3 (extru* or degenerat* or displac* or herniat* or prolaps* or sequestered or slipped or protru* or avuls*))).ti,ab \| \| 22. (coccy* adj2 (ach* or injur* or pain* or sprain* or strain*)).ti,ab \| \| 23. (coccygodyn* or coccalg* or coccygalg*).ti,ab \| \| 24. (lumbago* adj3 (pain* or symptom* or disabilit* or radiat* or leg* or spine* or spinal*)).ti,ab \| \| (piriformis* adj2 syndrome*).ti,ab \| \| 25. (sacr?* adj3 (pain* or facet* or (nerve adj2 root*) or osteoarth* or radicul* or stenos* or spondylo* or zygapophys* or injur* or discomfort* or dysfunction* or sore* or herniat*)).ti,ab \| \| 26. (sacr?* adj2 (ach* or injur* or pain* or sprain* or strain*)).ti,ab \| \| 27. ("si" adj2 (joint* adj3 (pain* or facet* or (nerve adj2 root*) or osteoarth* or radicul* or stenos* or spondylo* or zygapophys* or injur* or discomfort* or dysfunction* or sore* or herniat*))).ti,ab \| \| 28. (cervical* adj3 (dis?* adj3 (extru* or degenerat* or displac* or herniat* or prolaps* or sequestered or slipped or protru* or avuls*))).ti,ab \| \| 29. (cervical* adj3 (pain* or facet* or (nerve adj2 root*) or osteoarth* or radicul* or stenos* or spondylo* or zygapophys* or injur* or discomfort* or dysfunction* or sore* or herniat*)).ti,ab \| \| 30. (thoracic* adj3 (dis?* adj3 (extru* or degenerat* or displac* or herniat* or prolaps* or sequestered or slipped or protru* or avuls*))).ti,ab \| \| 31. (thoracic* adj3 (pain* or facet* or (nerve adj2 root*) or osteoarth* or radicul* or stenos* or spondylo* or zygapophys* or injur* or discomfort* or dysfunction* or sore* or herniat*)).ti,ab \| \| 32. (sciatic* adj3 (pain* or symptom* or disabilit* or radiat* or leg* or spine* or spinal*)).ti,ab \| \| 33. (stenos* adj2 (spine* or spinal* or vertebral*)).ti,ab \| \| 34. ((spine* or spinal*) adj2 osteoarthr*).ti,ab \| \| 35. (spinal* adj3 (condition* or diseas* or disabilit* or disorder* or degenerat* or pain* or stenos*)).ti,ab \| \| 36. (spine* adj3 (condition* or diseas* or disabilit* or disorder* or degenerat* or pain* or stenos*)).ti,ab \| \| 37. (spondyl* adj3 (condition* or diseas* or disabilit* or disorder* or degenerat* or pain*)).ti,ab \| \| 38. (radiculopath* adj3 (lumbar* or lumbo* or sacral* or sacro* or low-back* or lower-back* or cervical* or thoracic or spine* or spinal*)).ti,ab \| \| 39. (radiating* adj3 (lumbar* or lumbo* or sacral* or sacro* or low-back* or lower-back* or cervical* or thoracic or spine* or spinal*)).ti,ab \| \| 40. (radicular* adj3 (lumbar* or lumbo* or sacral* or sacro* or low-back* or lower-back* or cervical* or thoracic or spine* or spinal*)).ti,ab \| \| 41. (osteoarth* adj2 (knee* or hip*)).ti,ab \| \| 42. (musculoskeletal adj2 (pain* or disorder* or syndrome*)).ti,ab \| \| 43. (fibromyalgia adj3 (pain* or disorder* or disabilit* or diseas* or musculoskeletal* or symptom* or chronic)).ti,ab \| \| 44. (myofascial adj2 (pain* or syndrome*)).ti,ab \| \| 45. (tendinopath* adj2 (knee* or hip* or shoulder*)).ti,ab \| \| 46. (tendinit* adj2 (knee* or hips* or shoulder*)).ti,ab \| \| 47. (neck adj2 (pain* or injur* or syndrome* or disorder*)).ti,ab \| \| 48. shoulder* adj2 (pain* or injur*).ti,ab \| \| 49. knee* adj2 (pain* or injur* or syndrome* or disorder*).ti,ab \| \| 50. hip* adj2 (pain* or injur* or syndrome* or disorder*).ti,ab \| \| 51. patellofemoral adj2 (pain* or syndrome* or disorder*).ti,ab \| \| 52. shoulder adj2 (impingement* or syndrome* or disorder*).ti,ab \| \| 53. (rotator cuff* adj2 (syndrome* or diseas* or injur* or tear* or damage*)).ti,ab \| \| 54. persistent adj3 (pain* or syndrome*).ti,ab \| \| 55. pain adj2 (disorder* or syndrome* or musculoskeletal* or myofascial).ti,ab \| \| 56. (chronic adj2 (pain* or syndrome* or musculoskeletal)).ti,ab \| \| 57. longstanding pain*.ti,ab \| \| 58. (whiplash* adj2 (pain* or syndrome* or associated or disorder*)).ti,ab  59. or/ 1-58     \| 60. client education/ \| \| --- \| \| 61. health education/ \| \| 62. health literacy/ \| \| 63. health knowledge/ \| \| 64. health promotion/ \| \| 65. health information/ \| \| 66. psychoeducation/ \| \| 67. psychosocial rehabilitation/ \| \| 68. self-care/ \| \| 69. self-management/   \| 70. (patient adj2 (education* or advice or reassur*)).ti,ab \| \| --- \| \| 71. consumer adj2 health information*.ti,ab \| \| 72. (self* adj2 (care* or self-care*)).ti,ab \| \| 73. ((self* adj2 manag*) or self-manag*).ti,ab \| \| 74. ((self* adj help*) or self-help*).ti,ab \| \| 75. patient adj2 (empowerment*).ti,ab \| \| 76. (health adj2 (information* or literacy* or promotion)).ti,ab \| \| 77. (pain adj2 (education* or coping* or management*)).ti,ab \| \| 78. (therapeutic adj2 education).ti,ab \| \| 79. (explain adj2 pain).ti,ab \| \| 80. psychoeducation*.ti,ab \| \| 81. patient education* adj4 (online* or written* or oral* or verbal* or digital* or health*)).ti,ab \| \| 82. (education adj2 (neuroscience* or neurobiolog* or neurophysiolog*)).ti,ab \| \| 83. (patient education* adj4 (app or apps or application* or internet or website* or web-site* or web-base* or (web* adj2 page*) or (web* adj2 application*) or (web* adj2 interfac*))).ti,ab \| \| 84. (behavio?ral adj2 (education* or intervention*)).ti,ab \| \| 85. reassuring adj2 information*.ti,ab \| \| 86. (ergonomic* adj2 (education* or advice*)).ti,ab \| \| 87. (patient education* adj3 (individual or group* or group-based or face-to-face)).ti,ab  88. or/ 60-87     \| 89. "systematic review"/ \| \| --- \| \| 90. meta analysis/ \| \| 91. literature review/ \| \| 92. treatment guidelines/ \| \| 93. systematic* adj2 (review* or overview).ti,ab \| \| 94. meta?anal*.ti,ab \| \| 95. network meta?anal*.ti,ab \| \| 96. network meta anal*.ti,ab \| \| 97. (consensus adj2 (report* or statement)).ti,ab \| \| 98. (delphi adj2 (stud* or technique* or process* or consensus)).ti,ab \| \| 99. (guideline* adj (international or national)).ti,ab  100. or/ 89-99  101. 59 AND 88 AND 100  limit 101 to yr="2014 - 2024" \| \| \| \| \| |
| --- | --- | --- | --- | --- | --- | --- | --- | --- | --- | --- | --- | --- | --- | --- | --- | --- | --- | --- | --- | --- | --- | --- | --- | --- | --- | --- | --- | --- | --- | --- | --- | --- | --- | --- | --- | --- | --- | --- | --- | --- | --- | --- | --- | --- | --- | --- | --- | --- | --- | --- | --- | --- | --- | --- | --- | --- | --- | --- | --- | --- | --- | --- | --- | --- | --- | --- | --- | --- | --- | --- | --- | --- | --- | --- | --- | --- | --- | --- | --- | --- | --- | --- | --- | --- | --- | --- | --- | --- | --- | --- | --- | --- | --- | --- | --- | --- | --- | --- |
|  |
