# Supplementary material for: Patient education for chronic musculoskeletal pain: a scoping review of recommendations, effectiveness, and educational content
Source: Chiropr Man Therap. 2026 Jan 2;34:3. doi: 10.1186/s12998-025-00614-y (PMC12865995; doi:10.1186/s12998-025-00614-y)
Supplement: Supplementary file 2 — Supplementary Material 2 [file 12998_2025_614_MOESM2_ESM.docx]

Supplementary Table S1. Extracted information

| **Article Type** | **Data Extracted** |  |
| --- | --- | --- |
| All | Title, author, publication year  Design, study objective  Study or search period  Population: OA knee, OA hip, OA mixed, LBP, Neck pain, Spinal pain, Mixed chronic MSK pain  Target setting: Primary care, Secondary care, Physiotherapy, General practice, Mixed, No criteria Provider: Chiropractor, General practitioner, Medical doctors, Rheumatologist, Psychologist, Others, Mixed, No criteria.  Additional relevant information on patient education content. |  |
| Systematic reviews of primary studies and scoping reviews | Methodological Concerns (see Table 2)  Number of studies, total number of participants, countries/regions included. Education approach: Pain Neuroscience Education, Pain Coping Education, Cognitive Behavioral Education, Self-management Education, Narrative Education (based in patients' individual story), Condition Specific (Ergonomic or disease specific information), Other, Mixed, Undefined  Definition of educational approach Delivery Mode: Personal one-to-one, Personal group, Online one-to-one, Online group, Web/App resource (self-directed), Written material, Mixed, Other, Not reported  Dose: Span reported to be covered by included interventions.  Individualisation: No, Yes, some, Yes, highly individualised, Mixed, Undefined + describe:  If patient education was add-on to other intervention: Yes, No, Mixed, Undefined  Comparison: Any type of control, Usual care, No care, Exercise intervention, Other (describe).  Results, reported strength or certainty of evidence, sensitivity results, supported educational themes/messages. |  |
| Reviews of Clinical Practice Guidelines (CPG) | Methodological Concerns (see Table 2)  Number of CPGs, publishing organisations, CPGs geographical location.  CPGs aimed at: Monodisciplinary, Multidisciplinary, No specific target setting or profession. If monodisciplinary, write profession: e.g., Primary Care, Specialty or Secondary care, Physiotherapists, Chiropractors, General Practitioners, Rheumatologists, Other specialty/profession.  Overall recommendations regarding patient education.  Approach recommended: Pain Neuroscience Education, Pain Coping Education, Cognitive Behavioural Education, Self-management Education, Narrative Education (based in patients' individual story), Condition Specific (Ergonomic or disease specific information), Mixed, Other, Undefined.  Definition of educational approach.  Reported strength or certainty of evidence.  Specific themes recommended: Cause, Prognosis, Coping – general, Coping strategies - physical, Coping strategies – mental, Health care options, Other (self-) management options, Workplace / Return to work, Other.  Specific messages. |  |
| Delphi and consensus studies | Methodological Concerns (see Table 2)  Number of panellists first/last round: First round – Total, Researchers, Clinicians, Patients, Other stakeholders; Last round – Total, Researchers, Clinicians, Patients, Other stakeholders.  Professional backgrounds of experts: Chiropractor, General practitioner, Medical doctors, Rheumatologist, Psychologist, Others (write), Unknown.  Number of iterations.  Approach to education recommended: No recommendation of approach, Pain Neuroscience Education, Pain Coping Education, Cognitive, Behavioural Education, Self-management Education, Condition Specific (Ergonomic or disease specific information), Mixed, Other, Undefined.  Definition of educational approach.  Recommendation for dose: No, yes; If yes, describe.  Recommendations to individualise: No, yes; If yes, describe.  Theme recommended: Cause/Disease knowledge, Prognosis, Coping – general, Coping strategies - physical , Coping strategies – mental, Health care options, Other self-management options, Workplace / Return to work, Other.  Messages recommended, other recommendations regarding patient education, useful illustrations in paper. |  |
|  | | |

| Supplementary Table S2. Items considered in the critical appraisal of included studies | | |
| --- | --- | --- |
| Systematic Reviews | From AMSTAR 2: Explicit statement that the review methods were established prior to the conduct of the review; searched at least 2 databases;  searched study registries and grey literature; review authors performed study selection and data extraction in duplicate; used a satisfactory technique for assessing the risk of bias (RoB); provided a list of excluded studies with justification; meta-analyses justified the combination of data and used appropriate weighted technique; accounted for RoB in individual studies when interpreting the results; adequately investigated publication bias.  RoB items were not considered for scoping reviews. |  |
| Reviews of CPGs | Explicit statement that the review methods were established prior to the conduct of the review; searched at least 2 databases; searched other sources; review authors performed study selection and data extraction in duplicate; used a satisfactory technique for assessing the risk of bias (AGREE II or similar). |  |
| Delphi studies | Problem area clearly identified, selection of panel members based on objective and predefined criteria, anonymity of panellists during rounds, iterative rounds, defined consensus criteria, closing criteria defined a priori, stability of results^[[1]](#footnote-1)^. |  |
| All studies | “no serious concerns” could be noted even when one or more potential sources of bias were identified, if these were justified in the paper |  |
|  | |  |

Supplementary Table S3. Overview of systematic reviews on LBP or any spinal pain

| **Author, year** | **Number of included studies (Range)**  **Number of included patients (Range)** | **Number of systematic reviews investigating each outcome** |
| --- | --- | --- |
| **Pain Neuroscience Education** | | |
| Ma, 2024  Nunez-Cortes 2024  Nunez-Cortes, 2023  Puri, 2023*  Shin, 2023  Tegner, 2018  Wood, 2019 | 7 - 26 RCTs  300 - 1852 patients | Pain 7  Disability or activity limitation 6  Fear avoidance, kinesiophobia, catastrophizing 5  Knowledge, cognitions, beliefs 1  Self-efficacy 1  Psychological distress (depression, anxiety, stress) 1  Quality of life 2  Pain threshold 1  Physical function (e.g. strength, range of motion) 1  Sick leave 1 |
| **Mixed or undefined approaches** | | |
| Furlong 2022  Gomes 2024  Ho 2022  Jones 2021  Migliorini 2025  Piano 2025  Southerst 2023  Zahari 2020 | 5 - 97 RCTs  1132 - 13136 patients | Pain 8  Disability or activity limitation 8  Fear avoidance, kinesiophobia, catastrophizing 3  Knowledge, cognitions, beliefs 1  Self-efficacy 2  Psychological distress (depression, anxiety, stress) 3  Quality of life 5  Sick leave or return to work 2  Social participation 1  Global improvement 1 |

Supplementary Table S4. Overviews of systematic reviews on neck pain and whiplash associated disorders (WAD)

| **Author, year** | **Number of included studies (Range)**  **Number of included patients (Range)** | **Number of systematic reviews investigating each outcome** |
| --- | --- | --- |
| **Pain Neuroscience Education** | | |
| Lin, 2024  Palahi-Calsina 2025 | 7 RCTs  422 - 479 patients | Pain 2  Disability 1  Fear avoidance, kinesiophobia, catastrophizing 2  Knowledge, cognitions, beliefs 1  Self-efficacy 1  Physical function (e.g. strength, range of motion) 1 |
| **Mixed or undefined approaches** | | |
| Hainan, 2016 | 7 RCTs  7,022 patients (main part WAD) | Pain, Disability, Bothersomeness, Global improvement, Physical function, Emotional distress, Fear of reinjury, Stress, Perceptions of treatment efficiency and relief, Quality of life, Workdays lost, Medication use |

Supplementary Table S5. Overviews of systematic reviews on OA

| **Author, year** | **Number of included studies (Range)**  **Number of included patients (Range)** | **Number of systematic reviews investigating each outcome** |
| --- | --- | --- |
| **Pain Neuroscience Education** | | |
| Lesmond, 2024  Ordonez-Mora, 2022 | 4 - 7 RCTs 288 - 870 patients | Pain 2  Disability or activity limitation 1  Fear avoidance, kinesiophobia, catastrophizing 2  Quality of life 1 |
| **Self-management Education** | | |
| Kroon 2014  Salwana Kamsan, 2020*  Uritani 2021 | 7 -29 RCTs  1123 - 6753 patients | Pain 1  Disability or activity limitation 1  Self-management skills 1  Global OA scores 1  Quality of life 1  Engagement in life 1  Rate of withdrawals 1  Self-efficacy 1 |
| **Coping Skills Education** | | |
| Wang, 2021 | 10 RCTs  1195 patients | Pain 1  Disability or activity limitation 1  Fear avoidance, kinesiophobia, catastrophizing 1  Pain coping 1 |
| **Mixed or undefined approaches** | | |
| Goff, 2021  Isaji 2025  Sasaki, 2022 Sinatti, 2022  Simick Behera 2024 | 20 – 98 RCTs 2250 – 4107 patients | Pain 4  Disability or activity limitation 3  Fear avoidance, kinesiophobia, catastrophizing 1  Knowledge, cognitions, illness beliefs 1  Self-efficacy 2  Physical activity 1  Physical function (e.g. strength, range of motion) 1  Health care use 1 |

* The study was not included in the count of RCTs or outcomes as it only included RCTs with positive findings

Supplementary Table S6. Overviews of systematic reviews on mixed chronic MSK pain

| **Author, year** | **Number of included studies (Range)**  **Number of included patients (Range)** | **Number of systematic reviews investigating each outcome** |
| --- | --- | --- |
| **Pain Neuroscience Education** | | |
| Bülow 2021  Lepri 2023  Louw 2016  Romm 2020  Sanchez-Robalino 2025  Salazar-Méndez 2023  Salazar-Méndez 2023  Siddall 2022  Tatikola 2025  Watson 2019 | 5-24 RCTs  460 - 2352 patients | Pain 8  Disability or activity limitation 7  Psychological distress (depression, anxiety, stress) 5  Fear avoidance, kinesiophobia, catastrophizing 6  Knowledge, cognitions, illness beliefs 5  Self-efficacy 2  Hypervigilance 1  Pain threshold 2  Physical function (e.g. strength, range of motion) 1  Health care utilization 1  Pain coping 1  Quality of life 1 |
| **Mixed or undefined approaches** | | |
| Mullins 2022 | 4 RCTs  979 patients | Pain 1  Disability or activity limitation 1  Psychological distress (depression, anxiety, stress) 1  Self-efficacy 1  Coping strategies 1  Satisfaction 1 |

1. Nasa P, Jain R, Juneja D. Delphi methodology in healthcare research: How to decide its appropriateness. World J Methodol. 2021 Jul 20;11(4):116- [↑](#footnote-ref-1)
