# Supplementary material for: Patient education for chronic musculoskeletal pain: a scoping review of recommendations, effectiveness, and educational content
Source: Chiropr Man Therap. 2026 Jan 2;34:3. doi: 10.1186/s12998-025-00614-y (PMC12865995; doi:10.1186/s12998-025-00614-y)
Supplement: Supplementary file 7 — Supplementary Material 7 [file 12998_2025_614_MOESM7_ESM.docx]

**Content of Table 3 analysed by galaxy.ai thematic analysis generator 27-12-2024**

Prompt: What are the main themes that describe patients’ needs in relation to patient education?

No. 1 Thematic Analysis of Patient Needs in Relation to Patient Education

Main Themes and Sub-Themes

1. **Communication and Information Needs**

- **Clear Communication**: Patients expressed a strong need for clear, understandable communication from healthcare providers. This included avoiding medical jargon and using layman's terms.

- *Evidence*: Patients wanted explanations that were straightforward and relatable, as highlighted by comments about being "in the dark" regarding their conditions.

- **Personalized Information**: There was a consensus on the need for information tailored to individual circumstances, ensuring it was relevant and actionable.

- *Evidence*: Patients desired individualized care plans and found generic advice inadequate.

- **Multiple Sources of Information**: Patients sought information from various sources, including healthcare professionals, the internet, and support groups.

- *Evidence*: The reliance on the internet for health information was noted, with patients expressing difficulty in finding credible sources.

2. **Empathy and Understanding from Healthcare Providers**

- **Emotional Support**: Patients emphasized the importance of empathy and understanding from their healthcare providers.

- *Evidence*: Participants noted that they wanted to feel listened to and validated in their experiences, indicating that emotional connection is crucial for effective care.

- **Shared Decision-Making**: There was a strong desire for collaborative decision-making, where patients felt their opinions and preferences were respected.

- *Evidence*: Patients expressed a need for involvement in their treatment plans, seeking a two-way consultation process.

3. **Hope and Motivation**

- **Role of Healthcare Professionals**: Patients indicated that healthcare professionals play a vital role in fostering hope and motivation.

- *Evidence*: Participants sought reassurance and guidance from physiotherapists, highlighting the importance of health professionals in building self-efficacy.

- **Intrinsic and Extrinsic Motivations**: Factors influencing patient motivation included both personal desires for recovery and external encouragement from professionals.

- *Evidence*: The need for motivation was linked to behavior change and transitioning in healthcare practices.

4. **Understanding Pain and Its Management**

- **Complexity of Pain**: Patients recognized the multifaceted nature of chronic pain and expressed a desire for comprehensive explanations regarding its causes and management.

- *Evidence*: The importance of understanding the psychosocial aspects of pain was highlighted, with patients wanting to learn about the interplay between emotions and physical symptoms.

- **Self-Management Strategies**: There was a demand for information on self-management techniques to empower patients in handling their pain.

- *Evidence*: Patients wanted practical advice on managing flare-ups and returning to normal activities, reflecting a desire for autonomy in their care.

5. **Accessibility and Realism in Care**

- **Accessibility of Healthcare Services**: Patients expressed a need for healthcare services to be more accessible, similar to other long-term conditions.

- *Evidence*: The notion of a "health service merry-go-round" indicated frustration with repeated consultations without resolution.

- **Realistic Expectations**: Patients sought realistic information regarding their conditions, including prognosis and management strategies.

- *Evidence*: Participants wanted to understand what limitations they could exceed and how to actively engage in their recovery.

No. 2 Thematic Analysis of Patient Needs in Relation to Patient Education

Main Themes and Sub-Themes

1. **Communication and Information Needs**

- **Clarity and Understandability**

- Patients express a strong need for information that is clear, understandable, and devoid of medical jargon. They prefer explanations in "layman's terms" and require that communication be personalized to their individual circumstances.

- Evidence: “Patients wanted information that is understandable and informative, explained in ‘layman's terms.’” (Chou, 2018)

- **Empathy and Support**

- Patients seek empathetic communication from healthcare professionals (HCPs) and a sense of being listened to, which contributes to their overall satisfaction and engagement in the treatment process.

- Evidence: “The need for empathy, understanding and confidence.” (Chou, 2018)

- **Information Sources**

- Patients desire information from multiple sources, including healthcare providers, the internet, and peer experiences. They value credible and reliable sources to avoid conflicting advice.

- Evidence: “Patients typically relied on their doctor for general medical information… all participants stated they were keen to use the Internet as an alternative source of information.” (Chou, 2018)

2. **Patient Empowerment and Involvement**

- **Shared Decision-Making**

- The desire for shared decision-making is prominent, with patients wanting to be involved in their treatment plans and to have their preferences respected.

- Evidence: “Patients want to be listened to, believed and enabled to actively participate in a two-way consultation.” (Thompson, 2021)

- **Self-Management Strategies**

- Patients express a need for guidance on self-management strategies that empower them to take control of their health and pain management.

- Evidence: “Patients valued information that would help them deal with their back pain themselves.” (Lim, 2019)

3. **Understanding Pain and Its Implications**

- **Complexity of Pain**

- Patients acknowledge the complexity of pain and express a need for comprehensive explanations that encompass both physical and psychosocial factors.

- Evidence: “Patients want a medical diagnosis but also acknowledgment that psychosocial factors contributed to their pain.” (Chou, 2018)

- **Meaning and Personal Experience of Pain**

- Understanding the meaning of their pain is crucial for patients, as it affects their emotional well-being and engagement with treatment.

- Evidence: “Information is critical to help people understand and navigate the complex nature and meaning of pain.” (Thompson, 2021)

4. **Healthcare System Navigation**

- **Accessibility and Realism**

- Patients express the need for a healthcare system that is accessible and realistic, akin to the management of other chronic conditions.

- Evidence: “People living with chronic pain described needing a healthcare system that was accessible like other long-term conditions.” (Thompson, 2021)

- **Continuity of Care**

- The experience of being caught in a 'merry-go-round' of consultations highlights the need for continuity and coherence in care.

- Evidence: “The health service ‘merry-go-round,’ that is, continuous cycle of consultations repeating story.” (Thompson, 2021)

5. **Emotional and Psychological Support**

- **Hope and Reassurance**

- Patients seek hope and reassurance from healthcare professionals, emphasizing the importance of a supportive environment in their treatment journey.

- Evidence: “Hope is really important—the power of health professionals.” (Thompson, 2021)

- **Coping Mechanisms**

- The need for coping strategies is evident, as patients look for ways to manage the emotional toll of chronic pain.

- Evidence: “Patients wanted to know about self-management, i.e., what they could do about the pain and future treatment plan.” (Lim, 2019)

No. 3 Thematic Analysis of Patient Needs in Relation to Patient Education

Main Theme 1: Need for Effective Communication

- **Sub-theme 1.1: Clarity and Understandability**

- Patients expressed a strong desire for information that is clear and presented in layman's terms. They wanted explanations that are devoid of medical jargon, making it easier to understand their conditions and treatment options.

- Evidence: "Patients want information that is understandable and informative, explained in 'layman's terms.'"

- **Sub-theme 1.2: Personalized Communication**

- Patients preferred communication styles that were encouraging and tailored to their individual circumstances. They sought healthcare providers who actively listened and engaged in a two-way conversation.

- Evidence: "Participants want to be listened to, believed and enabled to actively participate in a two-way consultation."

#### Main Theme 2: Desire for Comprehensive Information

- **Sub-theme 2.1: Diagnosis and Understanding of Pain**

- Patients emphasized the importance of receiving a clear diagnosis and understanding the underlying causes of their pain. They viewed this as essential for validating their distress and improving treatment outcomes.

- Evidence: "Patients wished to find out about the problem, the prognosis, and how to prevent or manage recurrence."

- **Sub-theme 2.2: Management Strategies**

- There was a significant need for information regarding pain management, self-management strategies, and preventive measures. Patients expressed a desire to know what they could do to alleviate their symptoms and improve their condition.

- Evidence: "Patients valued information that would help them deal with their back pain themselves and were prepared to make behavioral changes."

#### Main Theme 3: Emotional and Psychological Support

- **Sub-theme 3.1: The Role of Hope and Reassurance**

- Patients sought hope and reassurance from healthcare providers. They valued the role of physiotherapists and other professionals in coaching and motivating them, which contributed to their self-efficacy in managing pain.

- Evidence: "Hope is really important—the power of health professionals."

- **Sub-theme 3.2: Acknowledgment of Emotions**

- Chronic pain is accompanied by complex emotions, and patients appreciated a supportive environment that allowed them to express their thoughts and feelings. They desired healthcare settings that fostered relaxation and a sense of belonging.

- Evidence: "Healthcare environments that promoted feelings of relaxation, a sense of belonging, and a safe environment to express thoughts and emotions were reported to be encouraging."

#### Main Theme 4: Accessibility and Continuity of Care

- **Sub-theme 4.1: Navigating the Healthcare System**

- Patients expressed frustration with the ‘merry-go-round’ of consultations, indicating a need for a more streamlined and accessible healthcare experience. They wanted a system that was as accessible as other long-term conditions.

- Evidence: "People living with chronic pain described needing a healthcare system that was accessible like other long-term conditions."

- **Sub-theme 4.2: Integration of Care**

- There was a need for integrated care that considers both the physical and psychosocial aspects of pain. Patients wanted healthcare providers to address both medical diagnoses and the emotional factors contributing to their pain.

- Evidence: "Patients wanted a medical diagnosis but also acknowledgment that psychosocial factors contributed to their pain."

#### Main Theme 5: Information Sources and Credibility

- **Sub-theme 5.1: Seeking Information from Multiple Channels**

- Patients utilized various sources for health information, including the internet, healthcare providers, and peer support. They valued credible and reliable information that could guide their understanding and management of pain.

- Evidence: "Patients typically relied on their doctor for general medical information, but once diagnosed with OA, all participants stated they were keen to use the Internet as an alternative source of information."

- **Sub-theme 5.2: Trust and Credibility of Information**

- Patients expressed concerns about conflicting advice from different healthcare practitioners, indicating a need for trustworthy and consistent information from their providers.

- Evidence: "Participants felt there was a contradiction in the advice and information given to some participants by various health care practitioners."

No. 4 Thematic Analysis of Patient Needs in Relation to Patient Education

Themes and Sub-Themes

1. **Communication and Information Needs**

- **Clarity and Understandability**: Patients desire information that is clear, concise, and explained in layman's terms. They express dissatisfaction with medical jargon and seek communication that is emotionally supportive and tailored to their personal circumstances.

- *Evidence*: “Patients wanted clear, easy to understand information” (Chou, 2018).

- **Personalization of Information**: There is a strong need for individualized care plans and treatment information that resonates with the patient’s unique circumstances and experiences.

- *Evidence*: “Communication style should be encouraging and personalized to the individual” (Chou, 2018).

- **Access to Reliable Sources**: Patients seek credible and trusted sources for health information, particularly given the conflicting advice often found online.

- *Evidence*: “Sourcing relevant and credible health information from the Internet was difficult” (Chou, 2018).

2. **Empathy and Emotional Support**

- **Need for Empathy**: Patients value healthcare practitioners who demonstrate understanding and compassion regarding their pain experiences.

- *Evidence*: “Patients had a need for empathy, understanding and confidence” (Chou, 2018).

- **Hope and Reassurance**: Patients often seek hope and motivation from healthcare professionals, which plays a crucial role in their coping strategies.

- *Evidence*: “Hope is really important—the power of health professionals” (Thompson, 2021).

3. **Decision-Making and Involvement**

- **Shared Decision-Making**: Patients want to be actively involved in their care decisions, emphasizing the need for two-way consultations where their voices are heard.

- *Evidence*: “Participants want to be listened to, believed and enabled to actively participate” (Thompson, 2021).

- **Understanding Pain Management Options**: Patients express a desire for comprehensive information about treatment options, including the implications of their choices and the role of self-management.

- *Evidence*: “Patients wanted to know about self-management, i.e., what they could do about the pain and future treatment plan” (Lim, 2019).

4. **Complexity of Pain and Health Conditions**

- **Understanding Pain**: Patients seek explanations about the nature of their pain, including the biopsychosocial factors that contribute to their condition.

- *Evidence*: “Patients wished to find out about the problem, the prognosis, and how to prevent or manage recurrence” (Chou, 2018).

- **Navigating the Healthcare System**: The experience of chronic pain often leads to frustration with the healthcare system, described as a ‘merry-go-round’ of consultations without resolution.

- *Evidence*: “The health service ‘merry-go-round’, that is, continuous cycle of consultations repeating story” (Thompson, 2021).

5. **Self-Management and Empowerment**

- **Encouragement of Self-Care**: There is a strong emphasis on patients wanting to learn self-management strategies and being empowered to take control of their health.

- *Evidence*: “Patients valued information that would help them deal with their back pain themselves” (Chou, 2018).

- **Motivating Factors**: Patients are motivated by intrinsic and extrinsic factors, including the desire for better health outcomes and support from technology and other resources.

- *Evidence*: “Motivating factors—intrinsic and extrinsic motivations, incentives, technology” (Thompson, 2021).

No. 5 Thematic Analysis of Patient Needs in Relation to Patient Education

Main Themes and Sub-Themes

1. **Communication and Information Needs**

- **Clarity and Understandability**: Patients express a strong need for health information to be communicated clearly and in layman's terms. They seek information that is understandable, avoiding complex medical jargon (Chou, 2018; Lim, 2019).

- Evidence: Patients reported feeling "in the dark" about their conditions and desired straightforward explanations of their diagnoses and treatment options.

- **Personalization**: The communication style should be tailored to individual patients, reflecting their unique circumstances and preferences (Chou, 2018).

- Evidence: Patients emphasized the importance of personalized communication regarding their pain management plans.

2. **Empathy and Support from Healthcare Providers**

- **Emotional Understanding**: Patients need empathy and emotional support from healthcare professionals. They want to feel listened to and validated in their experiences (Thompson, 2021; Chou, 2018).

- Evidence: Participants described a desire for healthcare providers to acknowledge their distress and provide reassurance.

- **Building Trust**: The relationship between patients and healthcare providers is crucial. Patients want to feel that their concerns are taken seriously and that they can trust their providers (Chou, 2018).

- Evidence: Patients reported dissatisfaction when they felt their healthcare providers lacked understanding or provided conflicting information.

3. **Shared Decision-Making**

- **Involvement in Care**: Patients express a strong desire to be active participants in their treatment decisions. They want to engage in shared decision-making with their healthcare providers (Thompson, 2021; Lim, 2019).

- Evidence: Participants emphasized the importance of being listened to and having their preferences considered in the treatment plan.

- **Respect for Autonomy**: Patients wish for their autonomy to be respected, indicating a need for healthcare providers to facilitate informed choices (Thompson, 2021).

- Evidence: Patients expressed a desire for guidance that empowers them to manage their conditions effectively.

4. **Comprehensive and Reliable Information**

- **Need for Accurate Diagnosis**: Patients want a clear diagnosis to understand the cause of their pain and to guide management strategies (Lim, 2019; Chou, 2018).

- Evidence: Participants indicated that having a diagnosis helped validate their experiences and improve treatment outcomes.

- **Access to Resources**: There is a need for information regarding self-management strategies, support services, and educational resources about their conditions (Lim, 2019; Chou, 2018).

- Evidence: Participants reported seeking information from various sources, including the internet, but struggled to find credible and relevant resources.

5. **Hope and Motivation**

- **Role of Healthcare Professionals**: Patients seek hope and motivation from their healthcare providers, viewing them as key figures in their recovery journey (Thompson, 2021).

- Evidence: Participants highlighted the importance of physiotherapists in fostering self-efficacy and encouraging behavior change.

- **Encouragement of Self-Management**: Patients desire information that empowers them to take control of their health and manage their pain effectively (Lim, 2019).

- Evidence: Participants expressed a willingness to engage in behavioral changes to alleviate their symptoms.

No. 6 Thematic Analysis of Patient Needs in Relation to Patient Education

Main Theme 1: Need for Effective Communication

- **Sub-theme 1.1: Clarity and Understandability**

- Patients expressed a strong desire for information that is clear and presented in layman's terms. They wanted explanations that are devoid of medical jargon, making it easier to understand their conditions and treatment options.

- Evidence: "Patients want information that is understandable and informative, explained in 'layman's terms.'"

- **Sub-theme 1.2: Personalized Communication**

- Patients preferred communication styles that were encouraging and tailored to their individual circumstances. They sought healthcare providers who actively listened and engaged in a two-way conversation.

- Evidence: "Participants want to be listened to, believed and enabled to actively participate in a two-way consultation."

#### Main Theme 2: Desire for Comprehensive Information

- **Sub-theme 2.1: Diagnosis and Understanding of Pain**

- Patients emphasized the importance of receiving a clear diagnosis and understanding the underlying causes of their pain. They viewed this as essential for validating their distress and improving treatment outcomes.

- Evidence: "Patients wished to find out about the problem, the prognosis, and how to prevent or manage recurrence."

- **Sub-theme 2.2: Management Strategies**

- There was a significant need for information regarding pain management, self-management strategies, and preventive measures. Patients expressed a desire to know what they could do to alleviate their symptoms and improve their condition.

- Evidence: "Patients valued information that would help them deal with their back pain themselves and were prepared to make behavioral changes."

#### Main Theme 3: Emotional and Psychological Support

- **Sub-theme 3.1: The Role of Hope and Reassurance**

- Patients sought hope and reassurance from healthcare providers. They valued the role of physiotherapists and other professionals in coaching and motivating them, which contributed to their self-efficacy in managing pain.

- Evidence: "Hope is really important—the power of health professionals."

- **Sub-theme 3.2: Acknowledgment of Emotions**

- Chronic pain is accompanied by complex emotions, and patients appreciated a supportive environment that allowed them to express their thoughts and feelings. They desired healthcare settings that fostered relaxation and a sense of belonging.

- Evidence: "Healthcare environments that promoted feelings of relaxation, a sense of belonging, and a safe environment to express thoughts and emotions were reported to be encouraging."

#### Main Theme 4: Accessibility and Continuity of Care

- **Sub-theme 4.1: Navigating the Healthcare System**

- Patients expressed frustration with the ‘merry-go-round’ of consultations, indicating a need for a more streamlined and accessible healthcare experience. They wanted a system that was as accessible as other long-term conditions.

- Evidence: "People living with chronic pain described needing a healthcare system that was accessible like other long-term conditions."

- **Sub-theme 4.2: Integration of Care**

- There was a need for integrated care that considers both the physical and psychosocial aspects of pain. Patients wanted healthcare providers to address both medical diagnoses and the emotional factors contributing to their pain.

- Evidence: "Patients wanted a medical diagnosis but also acknowledgment that psychosocial factors contributed to their pain."

#### Main Theme 5: Information Sources and Credibility

- **Sub-theme 5.1: Seeking Information from Multiple Channels**

- Patients utilized various sources for health information, including the internet, healthcare providers, and peer support. They valued credible and reliable information that could guide their understanding and management of pain.

- Evidence: "Patients typically relied on their doctor for general medical information, but once diagnosed with OA, all participants stated they were keen to use the Internet as an alternative source of information."

- **Sub-theme 5.2: Trust and Credibility of Information**

- Patients expressed concerns about conflicting advice from different healthcare practitioners, indicating a need for trustworthy and consistent information from their providers.

- Evidence: "Participants felt there was a contradiction in the advice and information given to some participants by various health care practitioners."

Overview of themes and sub-themes

**Communication and Information Needs**

- Clear Communication

- Clarity and Understandability

- Personalized Communication

- Personalized Information

**Desire for Comprehensive Information**

- Diagnosis and Understanding of Pain

- Need for Accurate Diagnosis

**Empathy and Understanding from Healthcare Providers / Emotional and Psychological Support**

- Emotional Support

- Empathy and Support

- **Coping Mechanisms**

- **Building Trust**

- ** Acknowledgment of Emotions**

****Hope and Motivation****

- **Intrinsic and Extrinsic Motivations**

- ** The Role of Hope and Reassurance**

**Respect for Autonomy**:

****Understanding Pain and Its Management****

- **Understanding Pain and Its Implications**

- **Complexity of Pain**

- **Meaning and Personal Experience of Pain**

**Understanding Pain Management Options**:

- **Complexity of Pain**

**Complexity of Pain and Health Conditions**

**Understanding Pain**:

- ** Management Strategies

****Patient Empowerment and Involvement****

- **Shared Decision-Making**

- **Decision-Making and Involvement**

- **Self-Management Strategies**

**Self-Management and Empowerment**

**Encouragement of Self-Care**

****Accessibility and Realism in Care** /**Healthcare System Navigation**/ Accessibility and Continuity of Care**

- **Accessibility and Realism**

- **Accessibility of Healthcare Services**:

- **Realistic Information**

- **Information Sources**

- **Continuity of Care**

**Navigating the Healthcare System**:

- ** Integration of Care**

**Information Sources and Credibility**

**Multiple Sources of Information**- difficulty in finding credible sources.

**Trust and Credibility of Information**

- ** Seeking Information from Multiple Channels**
